# A ZEB1-Neon knock-in uncovers traceable dynamics of epithelial-mesenchymal transition in tumors in vivo

**DOI:** 10.1186/s12915-026-02629-0

**Published:** 2026-05-12

**Authors:** Elisabetta D’Avanzo, Amelie Mahr, Nicolas Sauer, Simon Brandt, Ruthger van Roey, Harald Schuhwerk, Philipp Tripal, Benjamin Schmid, Stefanie Brey, Thomas H. Winkler, Simone Brabletz, Thomas Brabletz, Marc P. Stemmler

**Affiliations:** 1 Department of Experimental Medicine 1, Nikolaus-Fiebiger Center for Molecular Medicine, Friedrich-Alexander University of Erlangen-Nürnberg (FAU), Glückstr. 6, 91054 Erlangen, Germany; 2 Department of Dermatology, University Hospital Regensburg, Regensburg, Germany; 3 Optical Imaging Competence Centre Erlangen (OICE), Friedrich-Alexander University of Erlangen-Nürnberg (FAU), Erlangen, Germany; 4 Division of Genetics, Department of Biology, Friedrich-Alexander University of Erlangen-Nürnberg (FAU), Erlangen, Germany; 5 Comprehensive Cancer Center Erlangen-EMN (CCC ER-EMN), Bavarian Cancer Research Center (BZKF), Erlangen, Germany

**Keywords:** Mouse embryogenesis, Gene targeting, Fluorescent protein gene tagging, CRISPaint, CRISPR/Cas9, EMT, DNA damage, ZEB1, Breast cancer, Pancreatic cancer

## Abstract

**Background:**

Progression and metastasis of solid cancers are orchestrated by activation of epithelial-mesenchymal transition (EMT) in the primary tumor. This process is typically restricted to a limited number of cells that acquire partial or hybrid EMT states to unleash cellular plasticity. Capturing such dynamic and often reversible events in vivo on the single cell level is hampered by the lack of proper labeling tools that yet often induce permanent staining persisting beyond transient EMT activation.

**Methods:**

To enable live-tracking of EMT in vivo, we utilized CRISPaint and homologous recombination to endogenously tag ZEB1, one key transcription factor to activate EMT during tumorigenesis. Using the bright fluorescent protein mNeonGreen, we generated ZEB1-Neon fusion knock-in alleles in MDA-MB-231 and MCF10A cells, as well as in mice.

**Results:**

We demonstrate that mNeonGreen fluorescence is suitable to faithfully report on ZEB1 expression in vitro over time, becomes properly upregulated by TGFβ, and allows separation of ZEB1hi and ZEB1lo cells to capture different cellular properties, e.g., handling of DNA damage. The fusion does not affect ZEB1 function as evident by proper EMT induction, embryogenesis, and tissue homeostasis when present homozygously. Moreover, introducing Zeb1-Neon into the KPC mouse model of pancreatic cancer permits tracking of ZEB1+ cells in precision-cut slices and time-lapse imaging of isolated tumor cells.

**Conclusions:**

In summary, we provide a versatile tool that allows precise detection and live cell imaging of EMT, which will help to more accurately decipher the role of EMT in tumor progression and to identify therapeutic agents that can specifically manipulate EMT for novel combination therapies.

**Graphical Abstract:**

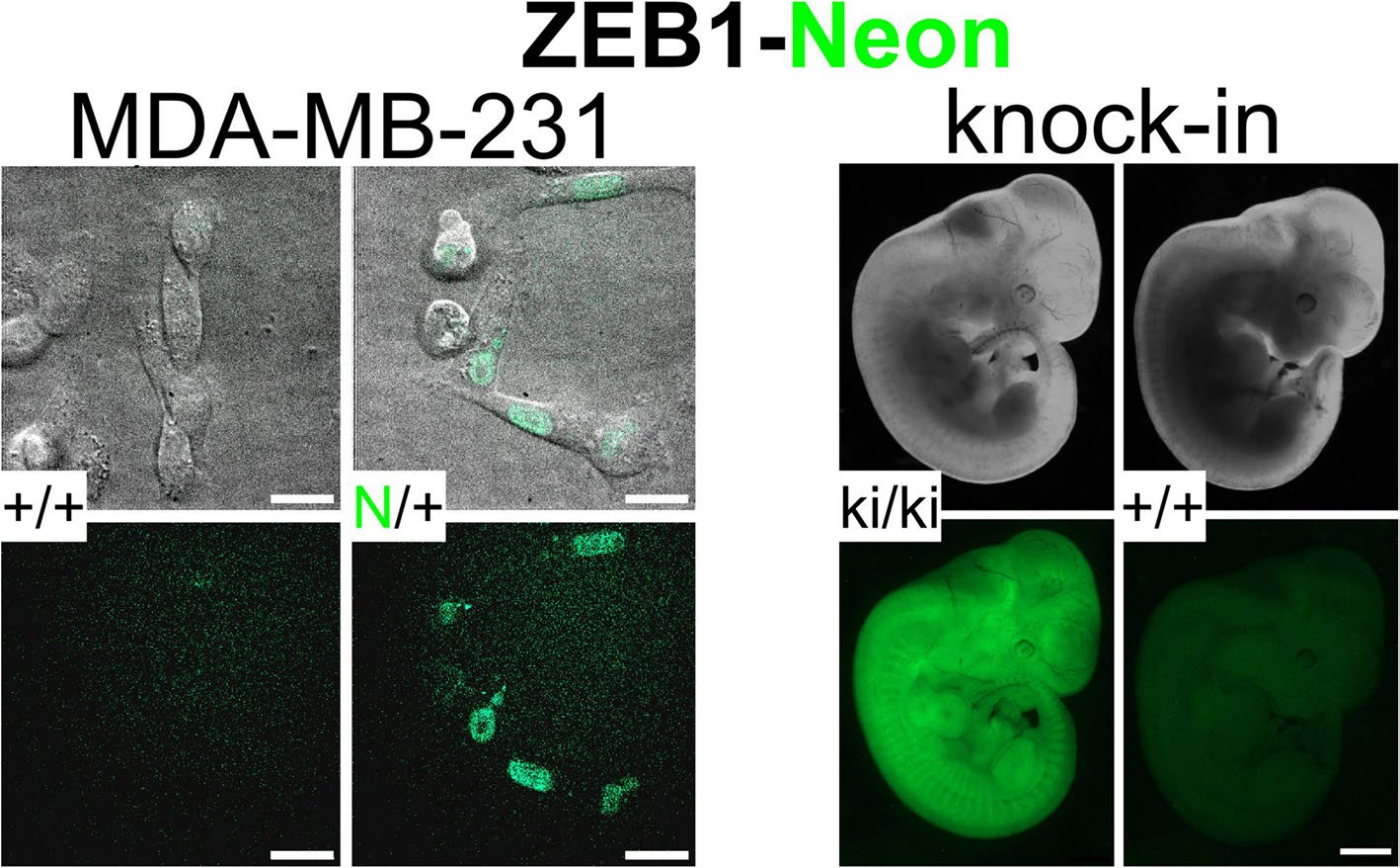

**Supplementary Information:**

The online version contains supplementary material available at 10.1186/s12915-026-02629-0.

## Background

The aberrant activation of epithelial-mesenchymal transition (EMT) is a key driver of tumor cell dissemination and metastasis, which activates cellular plasticity to allow cancer cells to adapt to changing conditions [1–5]. In many malignancies, ZEB1 is crucial to induce cell plasticity and to trigger EMT [6–9], thereby inducing tumor stemness, therapy resistance in favor of an undifferentiated aggressive tumor phenotype [2, 3, 5]. Due to acquired cellular plasticity and therapy resistance, such aggressive tumors are very refractory to standard treatment, demanding novel therapeutic approaches, e.g., to induce differentiation, block cell plasticity, or target de novo sensitivities [5]. Only recently it was identified that the EMT phenotype comes at the cost of increased vulnerability to the iron-dependent induced cell death of ferroptosis [10], regulated by ZEB1-dependent activity of key lipogenic enzymes to change the lipid membrane composition [11, 12]. Identifying ferroptosis inducers applicable in vivo and testing novel therapies options would benefit from detection of EMT events in vivo or ex vivo.

Detection of EMT in vivo has so far been hampered by the lack of reliable tools to report and to track such events in vivo. Permanent cell labeling is a valid option, but was challenged by identifying appropriate markers that can faithfully report on EMT and cell plasticity [13–16]. One recent approach utilized a CreERT2 knock-in strategy into *Tnc* and *Cdh2* loci, encoding two EMT-markers, that allows labeling of early and late EMT events in a spatio-temporal manner in the MMTV-PyMT mouse models of metastatic breast cancer. It revealed that partial EMT cells contribute to lung metastasis and chemoresistance, while full EMT cells mostly retain a mesenchymal phenotype incapable of lung colonization [17]. Contribution of early/late EMT events and partial EMT states to tumor progression was also found in similar approaches [17, 18], which can be exploited for drug screening [19]. To overcome limitations of indirect and non-transient labeling of EMT cells, endogenous tagging of key proteins involved in cell plasticity can provide a better spatio-temporal resolution of EMT.

Importantly, EMT and ZEB1 activation is a very dynamic process, activated mostly only transiently and locally in the tumor [20, 21]. Here, only a subset of tumor cells activates EMT, for example, when exposed to TGFβ. Moreover, even cells in culture show a very heterogenous pattern of ZEB1 expression [22]. Such populations can be divided into ZEB1-high (hi) and ZEB1-low (lo) cells that are different in their progression through the cell cycle, with ZEB1 accelerating S-phase entry, thereby triggering an adaptive DNA replication stress response engaging MRE11. This facilitates resilience to genotoxic stress by chemotherapy but renders ZEB1hi cells vulnerable to MRE11 inhibition [22].

Currently, our understanding of individual EMT events in vivo and the role of dynamically regulated ZEB1 in individual cells is hampered by the lack of tools to monitor and analyze such events. Is ZEB1 expression dynamically changing over time in steady state, upon EMT induction and during treatment in individual cells and how is the status propagated to daughter cells? The CRISPR/Cas9 technology led to development of various new techniques of gene editing, not only to inactivate or mutate gene loci, but also to easily label specific genes by molecular markers [23, 24]. The CRISPaint technology provides a very easy and efficient way to create such fusion proteins in cultured cells by fusing fluorescent protein and protein coding sequences at respective gene loci [25]. It allows to follow protein localization and expression dynamics in vivo in a spatio-temporal manner and was successfully used to tag, for example, E-cadherin (*Cdh1*), *Myh9*, and other cytosolic and transmembrane proteins in murine and human cell systems, including organoids [25–29]. Whether this method is also applicable to detect weakly expressed proteins like transcription factors has yet not been demonstrated.

We here show that endogenous tagging of ZEB1 with mNeonGreen (Neon) in cell lines and mice allows to observe ZEB1 dynamics and localization in vivo without interfering with ZEB1 function. The endogenous ZEB1-Neon fusion serves as traceable and reliable molecular surrogate of ZEB1 activity during embryogenesis as well as in cancer biology, demonstrated by high practicability and utility in various preclinical in vitro and in vivo models.

## Results

### Endogenous labeling of ZEB1 by mNeonGreen in living cells properly recapitulates ZEB1 expression in the breast cancer cell line MDA-MB-231

**Fig. 1:**
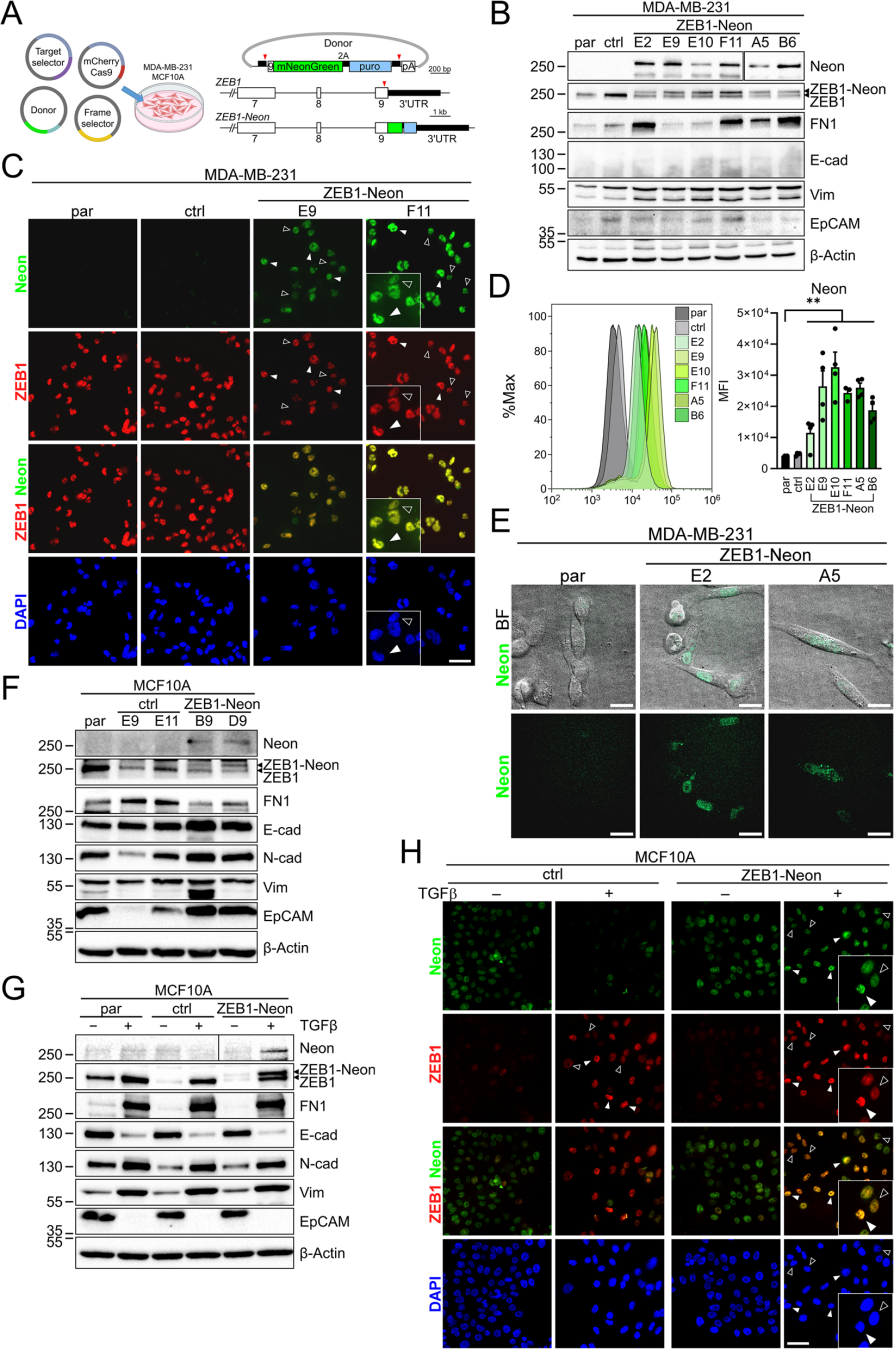
Endogenous tagging of ZEB1 by mNeonGreen (Neon) with CRISPaint faithfully reports on ZEB1 expression in MDA-MB-231 and MCF10A cells. **A** Schematic representation of the CRISPaint approach, using a modified donor plasmid for seamless fusion at *ZEB1* exon 9, integrating Neon-T2A-puro. sgRNA-directed expected Cas9 cuts in the *ZEB1* locus and plasmid are indicated by red arrowheads. **B** Western blot analysis of successfully targeted MDA-MB-231 after clonal expansion of ZEB1-Neon clones E2, E9, E10, F11, A5, and B6 together with the parental (par) and one control clone (ctrl). Anti-ZEB1 immunoblotting identifies both ZEB1 wildtype and ZEB1-Neon proteins as indicated. **C** Immunofluorescence staining (IF) using anti-mNeonGreen and anti-ZEB1 antibodies of selected clones from **B**. Nuclei are stained with DAPI; arrowheads, ZEB1hi cells; open arrow, ZEB1lo cells. **D** Representative FACS plots and mean fluorescence intensity (MFI) of *n* = 4 experiments for MDA-MB-231 ZEB1-Neon, par and ctrl clones as in **B**. **E** Detection of intrinsic ZEB1-Neon fluorescence and DIC (BF) imaging using spinning-disc confocal imaging of clones from **B**. **F**,** G** Western blot of MCF10A par, two ctrl (E9, E11), and two successfully CRISPaint-targeted clones (B6, D9) in standard culture (**F**) and upon TGFβ treatment for 14 days of par, F8 ctrl, and D9 Zeb1-Neon clones (**G**). For anti-Neon detection in **G**, samples were run on opposing ends of the same membrane and merged (vertical separation). **H** IF imaging of F8 ctrl and D9 MCF10A clones with and without TGFβ treatment for 14 days as in **C**; arrowheads, ZEB1hi cells; open arrow, ZEB1lo cells. Scale bars, 50 µm (**C**, **H**) and 20 µm (**E**)

To enable analysis of dynamic changes in ZEB1 expression in vivo, we applied the CRISPaint strategy [25] to introduce the coding sequence of mNeonGreen (Neon) 5′ of the stop codon into exon 9 of *ZEB1*. To this end, a donor plasmid was modified allowing excision and seamless fusion of a Neon-T2A-puro cassette including reintroduction of the otherwise lost last 17 amino acids due to sgRNA targeting (Fig. 1A). We first chose MDA-MB-231 cells due to their ZEB1-dependent EMT phenotype and high ZEB1 levels. Puromycin-resistant and Neon-positive cells were clonally expanded upon FACS separation and correct locus modification was confirmed by sequencing with efficiencies of 17% revealing exclusively heterozygous modifications (Additional file 1: Fig. S1A). Western blot and qRT-PCR analyses showed that specific ZEB1-Neon transcripts and protein were detected in all positive clones in contrast to the parental cell line and one control clone, demonstrating an expected size shift in anti-ZEB1 immunoblots (Fig. 1B, Additional file 1: Fig. S1B). Of note, similar band intensities of ZEB1 and ZEB1-Neon indicate that the Neon fusion did not alter ZEB1 expression levels and stability. Except for moderate variability of fibronectin (FN1) and EpCAM protein levels as well as *CDH1* (E-cadherin, E-cad) and *CDH2* (N-cadherin, N-cad) transcript levels (which are expressed close to detection limits), no major differences in EMT marker genes were observed in individual clones (Fig. 1B, Additional file 1: Fig. S1B). Although clonal variation in proliferation were observed, this was not correlated with ZEB1-Neon insertion (Additional file 1: Fig. S1C). Immunofluorescence staining confirmed expression of ZEB1-Neon (anti-Neon) exclusively in the nucleus that perfectly matched with total ZEB1 expression in location and intensity and was lost upon si*ZEB1* transfection (Fig. 1C, Additional file 1: Fig. S1D). Intrinsic ZEB1-Neon fluorescence was detected by FACS, demonstrating some clonal variation in the level of expression (Fig. 1D). Live cell confocal imaging allowed detection of ZEB1-Neon in vivo in high expressing cells (Fig. 1E, Additional file 2: Video S1).

Together, these results demonstrate that endogenous tagging of ZEB1 by Neon allows proper monitoring of ZEB1 in vitro and in vivo without affecting the cell phenotype.

### ZEB1-Neon is induced and follows the dynamics of TGFβ-mediated EMT in MCF10A cells

Next, we wondered whether ZEB1-Neon also follows dynamic regulation during EMT. We applied the CRISPaint approach to the well-established EMT model of nontumorigenic MCF10A cells. Successful integration of Neon-T2A-puro was confirmed by sequencing in 4% of analyzed clones (not shown), resulting exclusively in heterozygous targeting and detection of ZEB1-Neon at low levels in the untreated epithelial state by immunoblot (Fig. 1F). Treatment of MCF10A cells for 14 days with TGFβ resulted in loss of epithelial markers like E-cad and EpCAM, while mesenchymal markers like FN1, N-cad, and vimentin were induced. Importantly, ZEB1-Neon levels substantially increased after 14 days of TGFβ treatment resembling changes in ZEB1 (Fig. 1G, Additional file 1: Fig. S2A) that were also lost upon si*ZEB1* transfection (Additional file 1: Fig. S2B). The increase in ZEB1 that was mainly restricted to individual cells as demonstrated previously [22] was readily detectable by anti-Neon immunofluorescence, though with a higher background in control clones, but perfectly overlapping with anti-ZEB1 staining (Fig. 1H).

These data confirmed that ZEB1-Neon tagging in MCF10A cells can be used to track ZEB1 during EMT on a single cell level.

### In vivo Zeb1-Neon tagging efficiently reports on ZEB1 expression exemplified for mouse embryonic fibroblasts (MEFs)

**Fig. 2:**
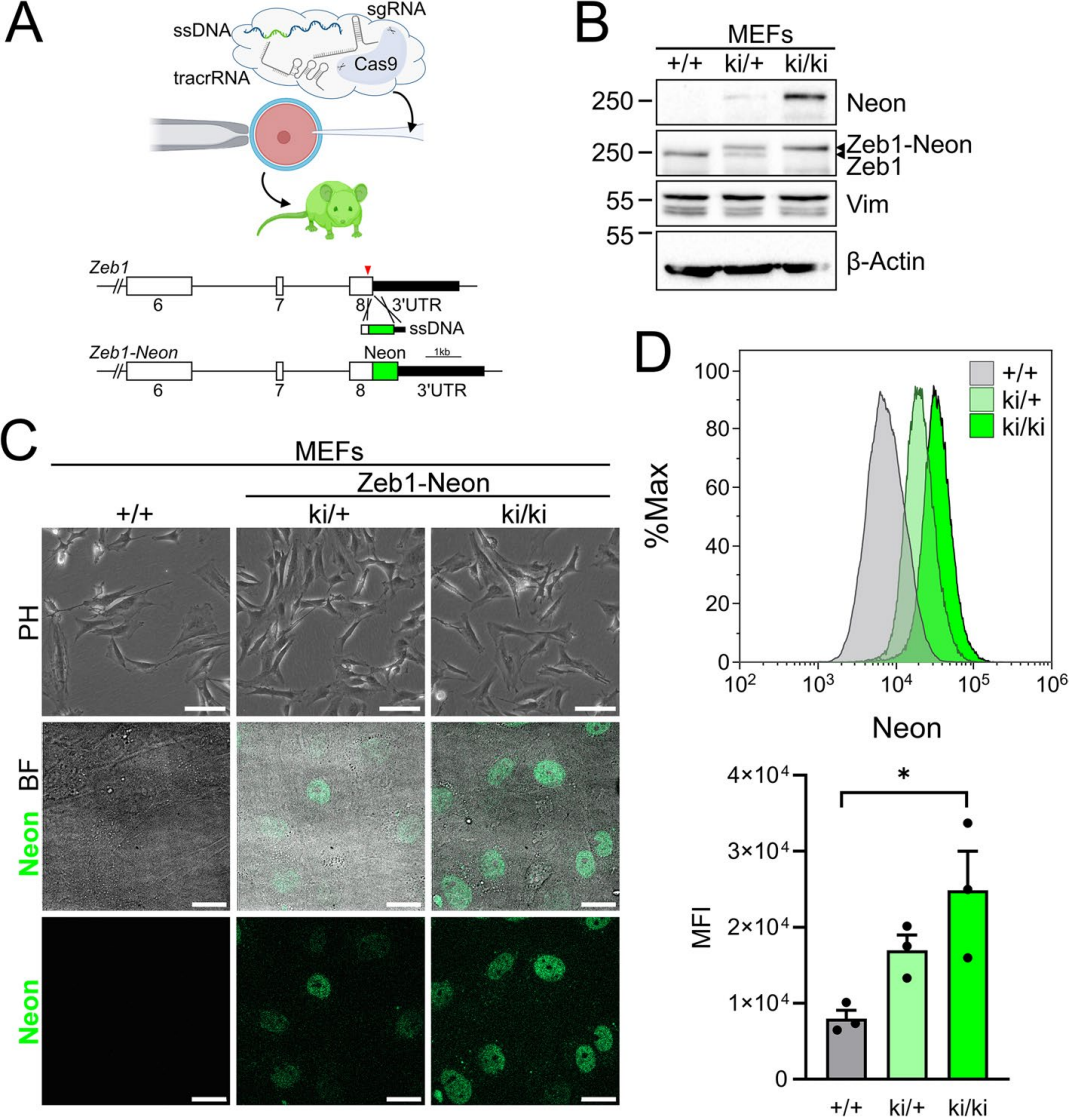
Mouse embryonic fibroblasts (MEFs) isolated from Zeb1-Neon mice after CRISPR/Cas9-mediated homologous recombination show robust detection of Neon. **A** Overview of oocyte injection and homologous recombination strategy using a ssDNA oligomer as template harboring the *Neon* coding sequence and short 5′- and 3′-homologous arms that was co-injected with Cas9 protein, specific gRNA, and tracrRNA into fertilized oocytes. **B** Sequence-confirmed founder mice (ki/+) were crossed inter se to generate MEFs with wildtype (+/+), heterozygous (ki/+), and homozygous (ki/ki) Zeb1-Neon genotypes, which were used for western blotting. Anti-Zeb1 specific immunoblot shows a molecular shift of Zeb1 and absence of a wildtype protein version in ki/ki MEFs. **C** Detection of intrinsic Neon fluorescence by spinning-disc confocal microscopy, demonstrating heterogenous Zeb1-Neon expression in a gene-dosage-dependent manner. **D** FACS analysis and MFI representation of Zeb1-Neon fluorescence in MEFs (*n* = 3)

To exclude that the fusion of Neon to ZEB1 affects ZEB1’s molecular function, we next sought to generate genome-edited mice by targeting Exon 8 of *Zeb1* for Neon insertion using microinjection of Cas9 protein together with guide and tracr RNAs and a single-strand (ss)DNA template into fertilized oocytes (Fig. 2A). The ssDNA oligo contained the Neon coding sequence and 170- and 270-bp up- and downstream regions, respectively. Resulting founder Zeb1-Neon knock-in (ki) mice were kept on a C57BL/6N background and evaluated for successful homologous recombination (11/20, 55%) and germline transmission of the ki allele (3/3, 100%) (Fig. 2A, Additional file 1: Fig. S3A). We first isolated embryonic fibroblasts (MEFs) from heterozygous intercrosses of wildtype (+/+), heterozygous (ki/+), and homozygous (ki/ki) E13.5 embryos. Similar to MDA-MB-231 and MCF10A cells, Zeb1-Neon was robustly detected by anti-Neon and anti-Zeb1 immunoblotting, demonstrating an expected partial or complete molecular shift from Zeb1 to the Zeb1-Neon fusion in ki/+ and ki/ki MEFs, revealing similar biallelic expression levels as Zeb1 +/+ MEFs (Fig. 2B). Of note, intrinsic Neon fluorescence was detected by live confocal microscopy, revealing lower and higher fluorescence intensity in mono- and biallelic knock-in configuration, while in both cases Zeb1hi and Zeb1lo cells could be identified (Fig. 2C). This effect of gene dosage was also reflected when comparing mean fluorescence intensities by flow cytometry (Fig. 2D). In line with moderate physiological Zeb1 levels in non-transformed cells, the overall mean fluorescence intensity in MEFs was lower than in the MDA-MB-231 breast cancer cells (ki/+ MEFs: 1.7 × 10^4^ and 0.8 × 10^4^ background MFI vs. MDA-MB-231: 2.5 × 10^4^ and 0.4 × 10^4^ background MFI).

In summary, the mouse Zeb1-Neon allele faithfully recapitulates Zeb1 expression in MEFs allowing label-free mono- and biallelic Zeb1 detection in vivo by intrinsic Neon fluorescence.

### Zeb1-Neon fusion preserves normal Zeb1 function allowing normal embryogenesis and adult life

**Fig. 3:**
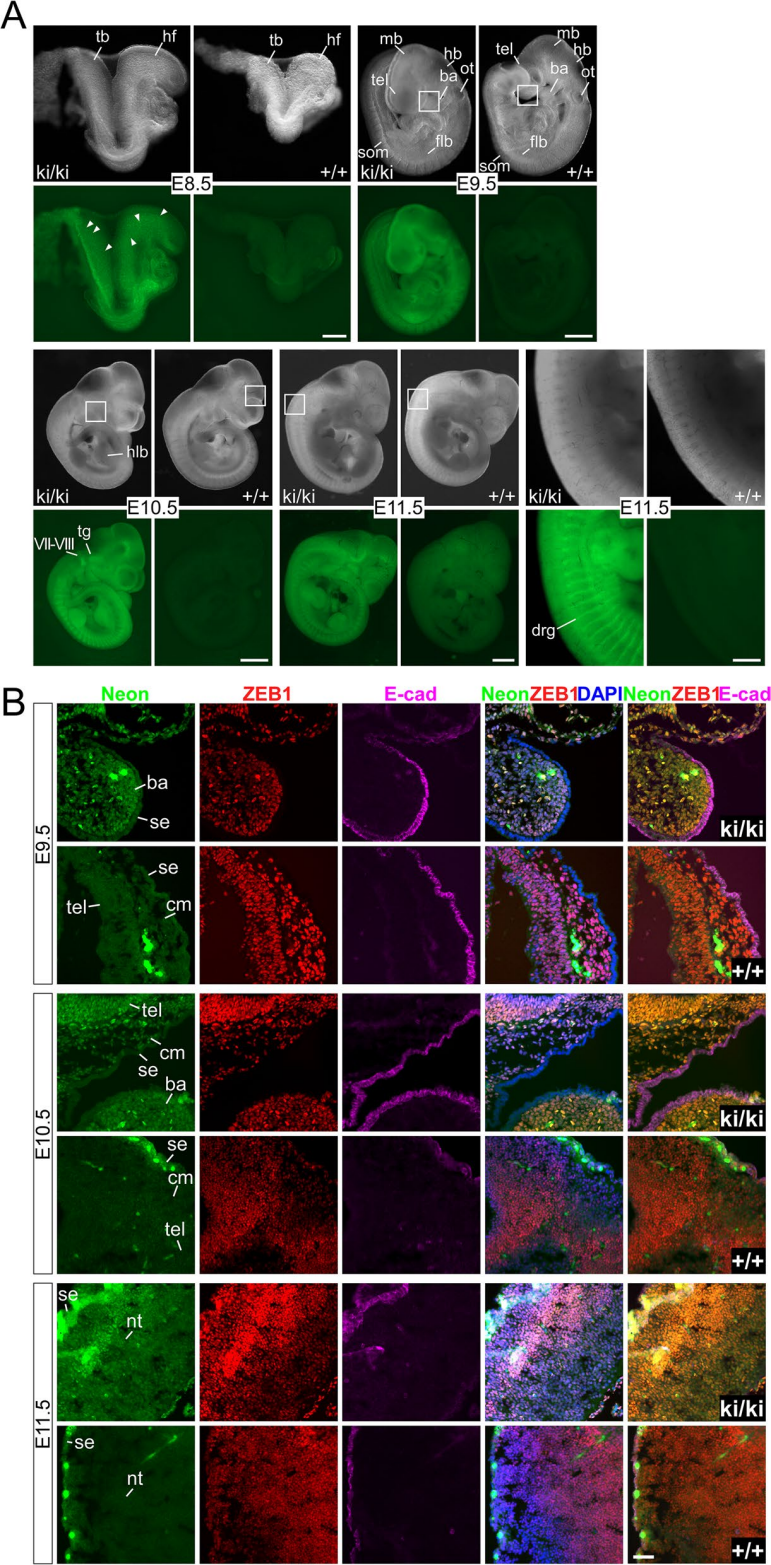
Detection of intrinsic Neon fluorescence in whole-mount embryos and on cryosections. **A** Isolated embryos between E8.5 and E11.5 imaged by stereomicroscopy directly after isolation, identifying green fluorescence exclusively in Zeb1-Neon embryos in specific regions as indicated; arrowheads, individual Neon+ nuclei. **B** Sagittal sections of cryoembedded embryos and IF labeling of Zeb1 (red) and E-cad (magenta), showing specific Zeb1-Neon nuclear detection in cells of mesenchymal and neuroectodermal origin, but not in epithelia in regions indicated in **A**, including branchial arch, telencephalon (E9.5, E10.5) and neural tube and surface ectoderm (E11.5). Spots of bright green fluorescence, also detected in all other channels and in +/+ embryos, represent autofluorescence from erythrocytes. Nuclei are stained with DAPI; ba, maxillary component of 2nd branchial arch; cm, cephalic mesenchyme; drg, dorsal root ganglion; flb, forelimb bud; hf, headfold; hlb, hindlimb bud; mb, midbrain; hb, hindbrain; nt, neural tube; ot, otic vesicle; se, surface ectoderm; som, somites; tel, telencephalon; tg, trigeminal ganglion; tb, tailbud; VII-VIII, facio-acoustic ganglia. Scale bars, 200 µm (E8.5), 500 µm (E9.5 and E11.5 zoom-in), 1 mm (E10.5 and E11.5), and 50 µm (**B**)

To further test normal function of Zeb1-Neon, we generated homozygous ki/ki embryos and offspring. Zeb1-Neon intrinsic fluorescence was readily detectable in unfixed E8.5 whole-mount embryos with a fluorescence stereomicroscope, revealing Zeb1 expression in headfolds, heart, somites, and tail buds (Fig. 3A, E8.5). At higher magnification, even individual Neon-positive nuclei could be discriminated in headfold and tail bud regions (Fig. 3A, E8.5, arrowheads). Neon intensity was increasing at E9.5 and next to the overall moderate intensity in the whole embryo several locations with increased expression were identified, including regions of the dorsal mid- and hindbrain, rhombomeres III and V, forelimb buds, and somites (presumably presumptive scleromyotome) (Fig. 3A, Additional file 1: Fig. S3B, E9.5). This pattern was changing during development, displaying a gradual loss of strong expression in the limb buds, with a more focused expression in dorsal root ganglia (Fig. 3A, Additional file 1: Fig. S3B, C, E10.5), most apparent at E11.5 during the phase of differentiation (Fig. 3A, Additional file 1: Fig. S3B, E11.5). Of note, the Neon expression pattern perfectly resembled the β-galactosidase activity in X-gal stained Zeb1-lacZ knock-in embryos, previously published [30] and are all in agreement with the known Zeb1 expression pattern, but with a much higher sensitivity allowing a more detailed observation with the new Zeb1-Neon allele. Zeb1-Neon embryos also consistently demonstrated different fluorescence intensities based on mono- and biallelic expression of the fusion protein (Additional file 1: Fig. S3B). Importantly, no defect or delay in embryogenesis/organogenesis was detected. In particular neither a curly tail neural tube closure defect, shortened stooped limbs, hemorrhages nor perinatal morbidity, reminiscent of *Zeb1* loss-of-function defects [30, 31], was observed. Proper Zeb1 function was also reflected in genotype distributions between E8.5 and E13.5, representing normal mendelian ratios (Additional file 1: Fig. S3D). Similarly, Zeb1-Neon ki/ki adult mice did not show any defects, pathological alterations, or changes in fertility in comparison to their littermates and were born in expected genotype distributions (Additional file 1: Fig. S3D). Zeb1-Neon intrinsic Neon fluorescence properly resembled Zeb1 immunolabeling by localization and intensity on cryosections of E9.5–E11.5 embryos. Expression was restricted to mesoderm and neurectoderm-derived tissues, including branchial arch and headfold mesenchyme, cells of the neural crest, forebrain, and neural tube, whereas it was absent in E-cad positive endoderm and ectoderm-derived cells (Fig. 3B). Increased expression of Zeb1-Neon in dorsal root ganglia was confirmed by increased Zeb1 immunoreactivity (Additional file 1: Fig. S3C). Similarly, specific Zeb1-Neon intrinsic fluorescence was detected in adult organs, which are much more prone to autofluorescence, in smooth muscle layers and the lamina propria of the small intestine (Additional file 1: Fig. S3E).

In summary, we conclude that Zeb1-Neon can functionally substitute Zeb1 wildtype protein under physiological conditions to allow proper embryogenesis and tissue homeostasis in adults, indicating that the fusion protein maintained Zeb1’s physiological functionality. Furthermore, Zeb1-Neon biallelic expression significantly increased fluorescence intensities, helping to better detect the protein during live cell imaging.

### ZEB1-Neon expression serves as a versatile tool to monitor and analyze ZEB1 expression dynamics of tumor cells 

**Fig. 4:**
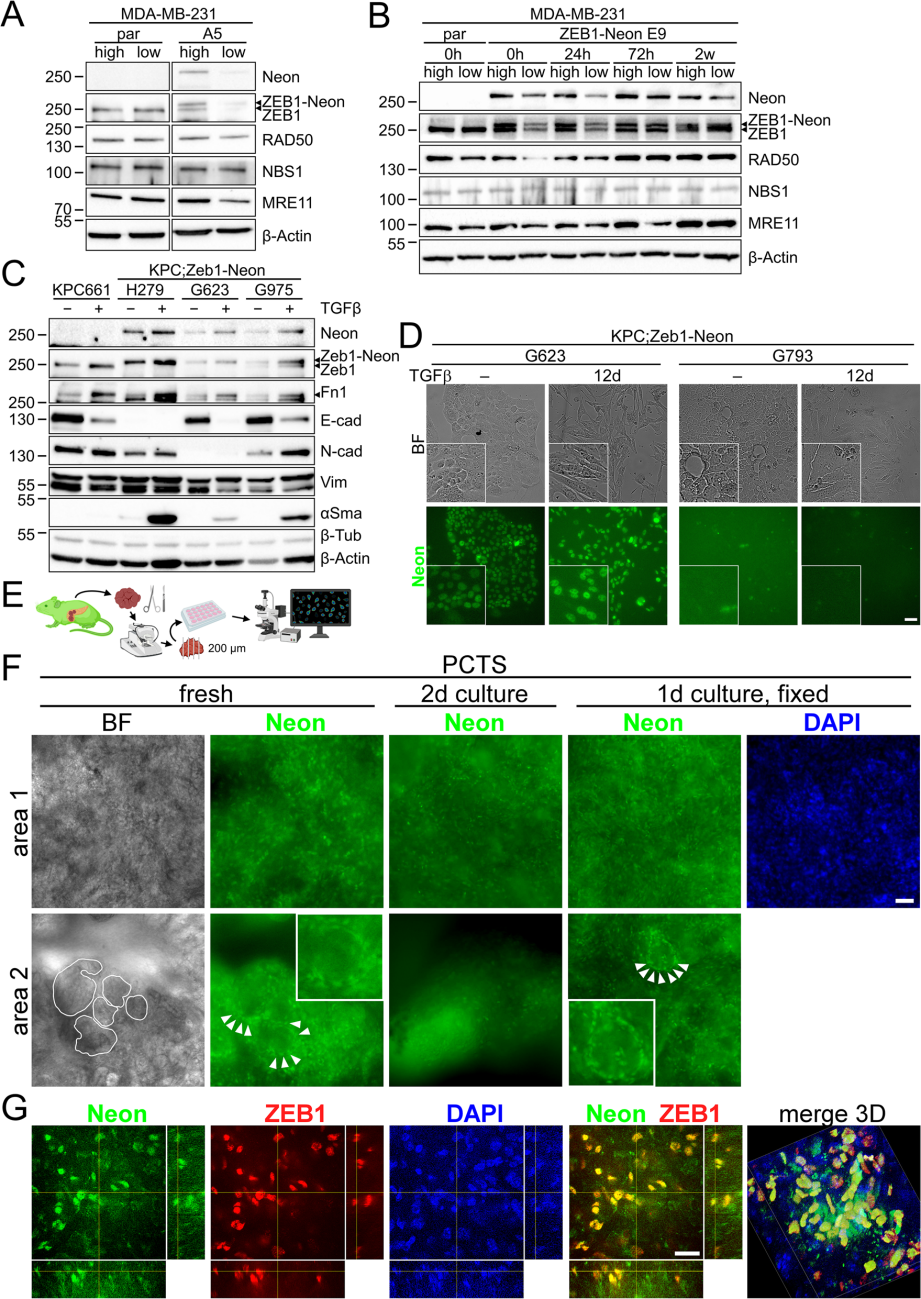
ZEB1-Neon/Zeb1-Neon alleles are suitable to analyze ZEB1 dynamics in cells and tumor PCTSs in vivo. **A** Separation of ZEB1hi and ZEB1lo cells of MDA-MB-231 ZEB1-Neon A5 cells by FACS and direct immunoblotting confirms different ZEB1 expression levels and ZEB1-dependent changes in expression of MRN complex components, which is not the case in parental (par) cells. **B** Time-course experiment following ZEB1hi and ZEB1lo cells after sorting of MDA-MB-231 parental and ZEB1-Neon E9 cells, analyzed directly after sorting (0 h) and during 2 weeks (2 w) of culturing, demonstrating the transient nature of ZEB1hi and ZEB1lo states. **C** Western blot analysis of KPC661 (epithelial) and KPC;Zeb1-Neon lines G623 (epithelial, ki/ki), H279 (mesenchymal, ki/ki), and G975 (epithelial, ki/+) showing changes in EMT marker expression in response to 8-day treatment with TGFβ. **D** Fluorescence in vivo imaging of two untreated epithelial KPC;Zeb1-Neon ki/ki cell lines (G623 and G793) and after 12-day TGFβ treatment using the EVOS system. Note that despite of the Zeb1-Neon ki/ki genotype in G793 cells, no Neon-specific fluorescence was detected, paralleling Zeb1 absence and lack of a TGFβ-mediated change in EMT marker expression. **E** Schematic representation of tumor dissection and PCTS generation from KPC;Zeb1-Neon tumors. **F** Fluorescence in vivo imaging of two regions (area 1, 2) of a PCTS generated from a KPC;Zeb1-Neon ki/ki tumor using the EVOS system, imaged directly after slice generation, after 2 days in culture and after fixation in 4% PFA. Note that tumor buds (white line) are Zeb1-Neon negative, surrounded by Zeb1-Neon positive stroma cells (arrowheads, area 2). Nuclei are stained with DAPI. **G** xyz projection and 3D reconstruction of a confocal z-stack of intrinsic Neon fluorescence and anti-Zeb1 IF labeling of a tumor PCTS. Nuclei are stained with DAPI. Scale bars, 50 µm (**D**, **F**) and 20 µm (**G**)

We tested whether the generated ZEB1-Neon fusion protein can also help to uncover dynamics in ZEB1 expression. Previously, we showed that ZEB1 in tumor cell cultures has a very heterogenous expression pattern [22]. Moreover, EMT cells in general and cells with high levels of ZEB1 (ZEB1hi) in particular have intrinsically different characteristics in terms of cell cycle progression, crosstalk with and dependency on the DNA damage response (DDR) compared to cells with low levels of ZEB1 (ZEB1lo) [32]. To test whether ZEB1hi cells retain high ZEB1 levels and propagate this to daughter cells or whether expression changes very dynamically, we used MDA-MB-231 ZEB1-Neon and parental cells and subjected them to FACS to isolate 5% top and bottom populations with highest and lowest Neon fluorescence, respectively (Additional file 1: Fig. S4A). Western blotting directly after sorting confirmed successful separation of ZEB1hi and ZEB1lo cells in different clones, whereas no such separation was observed in parental MDA-MB-231 cells after sorting of 5% high and low cells based on background fluorescence (Fig. 4A, Additional file 1: Fig. S4B). Strikingly, similar to previous observations, ZEB1hi cells showed moderately increased levels of RAD50, NBS1, and MRE11, indicative of increased MRN complex expression to cope with the ZEB1hi-mediated inflicted endogenous DNA replication stress [22] (Fig. 4A, Additional file 1: Fig. S4B, C). As expected, parental cells did not display altered MRN complex levels in agreement with the notion that background fluorescence is incapable of separating these different cellular features (Fig. 4A, B, Additional file 1: Fig. S4B, C). Interestingly, culturing of separated ZEB1hi and ZEB1lo cells showed that the difference in ZEB1(-Neon), RAD50, and MRE11 expression was maintained for 24–72 h after sorting, but led to a convergence in expression of both samples after 2–5 weeks in culture (Fig. 4B, Additional file 1: Fig. S4D, E), supporting that high phenotypic plasticity and not stable intrinsic differences are the cause of the hi/lo expression states.

In summary, ZEB1-Neon expression allows separation and isolation of live cells based on ZEB1 expression levels and downstream analysis helps to decipher different ZEB1-dependent cell characteristics. Moreover, we show that differences in ZEB1 levels in standard cultures are transient and are balanced out after several passages.

### Zeb1-Neon expression allows label-free detection of Zeb1 dynamics in tumor slice cultures and in cultured KPC cells by time-lapse imaging

Next, we addressed whether the endogenously tagged Zeb1-Neon knock-in allele would properly report on Zeb1 expression in mouse tumors. We crossed the Zeb1-Neon allele into the KPC model of pancreatic ductal adenocarcinoma (PDAC) [6, 33] to create KPC;Zeb1-Neon ki/ki and ki/+ mice (*Kras*^*LSL.G12D/+*^;*Tp53*^*LSL.H172R/+*^;Pdx1-cre;*Zeb1*^*ki/ki*^ and *Kras*^*LSL.G12D/+*^;*Tp53* ^*LSL.H172R/+*^;Pdx1-cre;*Zeb1*^*ki/*+^). We isolated KPC;Zeb1-Neon ki/ki cells with a mesenchymal phenotype (H279) (Additional file 1: Fig. S5A) with expected high levels of Zeb1. Intrinsic fluorescence of the fusion protein was analyzed by live cell imaging revealing robust and heterogenous Zeb1-Neon nuclear expression. No fluorescence was observed in *Zeb1*^+*/*+^ KPC701 cells (Fig. 4C) [6]. Intrigued by these findings, we used isolated epithelial-type PDAC lines G623 (KPC;Zeb1-Neon ki/ki), G793 (KPC;Zeb1-Neon ki/ki), and G975 (KPC;Zeb1-Neon ki/+) and subjected them to TGFβ treatment for 12 days to induce EMT. Except for G793, all lines showed a moderate upregulation of Zeb1(-Neon) and expected changes in EMT marker gene expression to varying degree, including Fn1 and αSMA upregulation in the mesenchymal H279 line (Fig. 4C, Additional file 1: Fig. S5B, C). Fluorescence imaging using an EVOS microscope demonstrated that robust Zeb1-Neon specific intrinsic fluorescence was already detected in untreated G623 cells which was restricted to the nucleus and further increased after 2 days and 12 days of TGFβ treatment (Additional file 1: Fig. S6A, Fig. 4D). Interestingly, no fluorescence signal was detected in G793 epithelial cells neither before nor after TGFβ exposure (Fig. 4D, Additional file 1: Fig. S6A). This was in line with undetectable Zeb1 expression and no response of EMT marker genes to TGFβ treatment (Additional file 1: Fig. S5B). Of note, Neon fluorescence of both H279 mesenchymal and G623 epithelial cell lines was detectable with standard inverted cell culture microscopy before and after TGFβ treatment (Additional file 1: Fig. S6B, C), whereas in control mesenchymal KPC701 and G793 cells only autofluorescence from dying or dead cells was observed. The analysis showed that introducing Zeb1-Neon in a homozygous configuration into the KPC model allows robust detection of Zeb1 dynamics in cultured tumor cells in vivo and separation of highly plastic (G975) from phenotypically fixed (G793) cancer cells.

To extend this analysis in KPC PDAC, we established precision-cut tissue slice (PCTS) cultures of a freshly isolated KPC;Zeb1-Neon ki/ki tumor (Fig. 4E). We found areas with round-shaped condensed, presumptive nuclear Zeb1-Neon fluorescence in a large proportion of cells in several z-planes directly after cutting (Fig. 4F, area 1 fresh, Additional file 2: Video S2). As expected, the majority of these Zeb1-Neon positive cells were found in areas surrounding bud- and gland-like structures which were Zeb1-Neon negative, suggesting Zeb1-positive stroma cells in proximity to epithelial Zeb1-negative tumor cells (Fig. 4F, area 2 fresh). Importantly, the fluorescence signal from Zeb1-Neon was maintained during 2 days of culture as well as after fixation of the PCTSs, indicating that Zeb1 expression was not globally changing during culture and integrity and survival of the tumor tissue was maintained (Fig. 4F, 2-day culture and fixed). The quality of standard fluorescence microscopy imaging of PCTSs suffers from out of focus fluorescence; therefore, we performed high-resolution confocal imaging. It confirmed intrinsic Neon fluorescence that overlapped with Zeb1 immunofluorescence labeling (Fig. 4G, Additional file 2: Video S3), overall confirming that Zeb1-Neon expression robustly reflects Zeb1 expression. Thus, our reporter allows to analyze Zeb1 dynamics in tumor PCTSs over time.

### Zeb1-Neon properly reports on temporal dynamics during EMT induction in tumor cells

In a final experiment, we addressed whether Zeb1 dynamics can be followed in a time-lapse approach in cultured cells in steady-state cultures and upon induction of EMT. We first monitored one Zeb1-Neon+ (G623) and one Zeb1-Neon − (G793) cell line for 4 days in 2-h intervals using a live cell imaging approach (Incucyte S3). Time-lapse imaging confirmed specific Zeb1-Neon detection exclusively in G623 and revealed very interesting Zeb1-dependent cell dynamics. In general, Zeb1hi and Zeb1lo cells were identified which dynamically switched between states over the 4-day period. We found clusters of cells that are characterized by very high Zeb1-Neon expression and extreme motility, interacting with several Zeb1lo cell clusters on the plate. These cells maintained a Zeb1hi state for at least 24 h (Additional file 1: Fig. S6D, G623, upper panel, Additional file 2: Video S4). In other clusters, cells with moderately elevated Zeb1 in less mobile colonies maintained this Zeb1-Neon expression level over a similar period, while the bulk population maintained a low Zeb1-Neon profile (Additional file 1: Fig. S6D, G623, middle panel, Additional file 2: Video S5). No specific fluorescence was observed in Zeb1-Neon negative cells (Additional file 1: Fig. S6D, G793, lower panel, Additional file 2: Video S6).

**Fig. 5:**
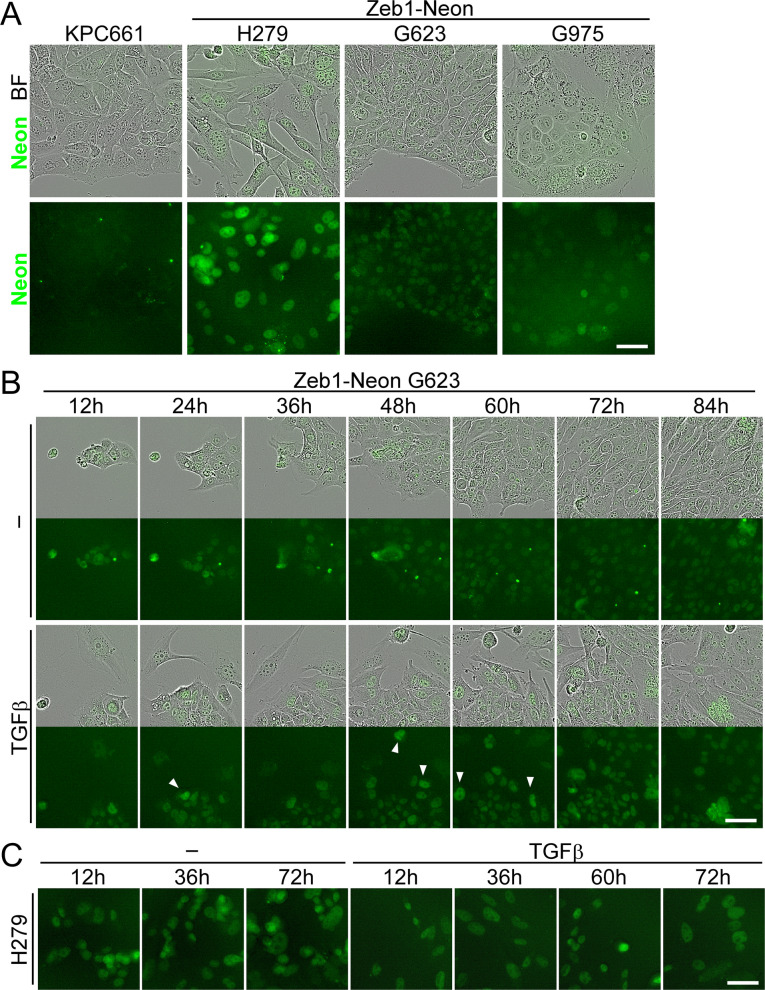
Zeb1-Neon allows detection of dynamic Zeb1 expression over time during TGFβ-mediated EMT. **A** Live cell imaging of steady-state culture of KPC661 and mesenchymal (H279) and epithelial (G623 ki/ki and G975 ki/+) KPC;Zeb1-Neon cells using Incucyte S3. **B** Still images of a 4-day TGFβ treatment and continuous live cell imaging of G623 cells showing increased Zeb1-Neon expression in response to TGFβ presented as overlay of bright field and Neon fluorescence (upper) and isolated green channel (lower panels); arrowheads, individual Zeb1hi cells. **C** Still images of a 4-day TGFβ treatment and continuous live cell imaging of mesenchymal H279 cells showing no change in Zeb1-Neon expression (green fluorescence channel) in response to TGFβ. Scale bars, 50 µm

We tested four KPC and KPC;Zeb1-Neon cell lines for changes in Zeb1-Neon specific fluorescence in response to TGFβ treatment during EMT induction. In agreement with previous analyses, KPC661 cells did not show Neon fluorescence (Fig. 5A, Additional file 2: Video S7), while among the three KPC;Zeb1-Neon lines the mesenchymal cell line H279 showed the highest and the epithelial lines G623 and G975 showed moderate and weak Zeb1-specific detection of nuclear Neon fluorescence in steady-state cultures, reflecting their bi- and monoallelic knock-in configuration, respectively (Fig. 5A). Over a period of 4 days, a gradual increase in Zeb1-Neon fluorescence was specifically observed in G623 and G975 cells when exposed to TGFβ, which first resulted in an increase of isolated Zeb1hi cells (Fig. 5B, arrowheads), followed by an overall increase within the whole population, but with a maintained Zeb1hi/Zeb1lo heterogeneity (Fig. 5B, Additional file 2: Videos S7–S10). When cells were pretreated for 6 days before starting live cell imaging (6–10-day TGFβ), G623 and G975 cells already showed higher levels of fluorescence at the beginning of recording, which did not increase further (Additional file 2: Videos S9 and S10). Using automated detection of single nuclei and measuring fluorescence intensities over time confirmed a TGFβ-mediated increase in intensity-sum Zeb1-Neon fluorescence and in the Zeb1hi population in 0–4-day TGFβ-treated cells, whereas ratios in 6–10-day TGFβ-treated cells stayed fairly constant on high levels over time (Additional file 1: Fig. S6E). In contrast, mesenchymal H279 cells did not show a detectable increase in Zeb1-Neon fluorescence over time (0–3-day and 6–10-day TGFβ), although they changed to a more mesenchymal morphology, in line with upregulation of Fn1 and αSMA (Figs. 4D, 5C, Additional file 2: Video S8). This experiment shows that Zeb1 upregulation in response to EMT triggers can be detected in individual cells and their fate and Zeb1-Neon expression dynamics can be followed over time.

## Discussion

Live cell imaging and identification of protein expression levels in individual cells in vivo is key to identify cell phenotypes that are correlated with specific signaling events. Fluorescently tagged proteins can help to analyze protein function in a spatio-temporal manner in vivo and how expression levels and subcellular localization influence cell properties. In particular, it allows to connect protein function with changes in cell phenotypes during dynamic processes like EMT, which induces controlled (not necessarily linear) expression dynamics over time [34–36]. In this context, endogenous labeling of EMT transcription factors like ZEB1 provides a versatile approach to follow expression dynamics during EMT in cancer or embryogenesis as well as for analyzing the basis of heterogeneous gene expression in steady-state cultures.

Due to the general low abundance of transcription factors, detection of endogenous fusion proteins with fluorescent proteins can be challenging, requiring bright photostable monomeric fluorescent proteins and low backgrounds. mNeonGreen fulfills such requirements in terms of brightness and stability in comparison to other green fluorescent proteins [37]. By using CRISPaint and Cas9-mediated homologous recombination in oocytes to endogenously tag ZEB1 with Neon in cell lines and mice, respectively, we show that ZEB1 can be visualized by live cell imaging. With this reliable tool, we found that dynamic changes of ZEB1 expression during TGFβ treatment can be detected by time-lapse microscopy and ZEB1hi and ZEB1lo cells can be separated for downstream analysis. We confirmed that expression of MRN complex components is directly correlated with ZEB1 levels [22] and these phenotypic differences are transient, as they converge upon long-term cultivation. Moreover, we demonstrate that Neon fluorescence can be utilized to isolate ZEB1-positive living cells which can be applied to a mix of different cell types, e.g., from whole organs or tumors, which is suitable for identifying ZEB1 function, for example, in subpopulations of macrophages [38] or fibroblasts [39]. Furthermore, Zeb1-Neon knock-in mice robustly report on ZEB1 expression in vivo for better understanding of its role in development as well as of its dynamic expression in several cell types during tumor progression using PCTS cultures of PDAC.

The tagging of ZEB1 was remarkably efficient without any detectable off-target events. Since MDA-MB-231 cells express robust levels of ZEB1, sorting of Neon+ cells yielded 17% of correctly targeted events and even isolation of non-sorted transfectants in MCF10A cells resulted in 4% efficiency of correct gene targeting. In mice, Cas9-induced homology-directed repair even revealed a > 50% success rate upon oocyte injection. This high efficiency allows to easily apply this approach to other genes and even use combinations of multiple protein labeling in the same cell or mouse line [26, 27]. Moreover, the specific loss of Neon fluorescence upon si*ZEB1* knockdown in MDA-MB-231 and MCF10A cells and the very precise ZEB1-matched pattern of Neon fluorescence in embryos that was consistently maintained over several generations of backcrossing provide indirect evidence that the design of sgRNAs did not result in off-target events.

During our study, we made very interesting observations. First, we found that isolated ZEB1hi and ZEB1lo cells indeed show higher and lower expression of the MRN complex, respectively, and that these states are re-converging over time when cultivated separately over several passages, presumably re-establishing an advantageous ZEB1hi-lo population equilibrium. Second, in KPC;Zeb1-Neon tumor cells, a Zeb1hi state can persist for at least 24 h and an extraordinary high Zeb1 level can exist in clusters of cells that then show exceptionally high motility during migration, involving transient interaction with other tumor cells. This observation may not only explain and confirm the process of cell migration/invasion during EMT and metastasis, but also provide new insights into how partial or full EMT cells interact and communicate with the stroma and other tumor cells to foster dissemination and metastatic colonization.

Future applications of this ZEB1 reporter include higher-resolution time-lapse imaging of various ZEB1-positive cells and intra-vital imaging by two-photon microscopy. In addition, drug screens for changes in ZEB1-Neon fluorescence intensity will help to identify substances that promote mesenchymal-epithelial transition (MET), EMT, or act plastistatic [5]. ZEB1-Neon can also be combined with successfully established EMT sensors, like *CDH1* and *VIM* driven reporters [19] or with ferroptosis/lipid-reactive oxygen species (lipid-ROS) sensors [11]. In addition, the ZEB1-Neon reporter may help to decipher the specific and context-dependent roles of specific DNA damage responses, such as DNA double-strand break (DSB) repair pathway choice that is co-regulated by ZEB1 [32]. For example, on one hand, in breast cancer ZEB1 was shown to inhibit the error-prone microhomology-mediated end joining (MMEJ) by repressing Polθ [40] in favor of non-homologous end joining (NHEJ) to suppress homologous recombination (HR) by recruiting 53BP1 to DSBs [41]. On the other hand, it was demonstrated that during the acquisition of radioresistance, ZEB1 elevates ATM levels to promote HR [42, 43]. Moreover, the DSB repair pathway choice depends on cell-cycle phase-determined regulation of DNA end resection and is strongly impacted by TGFβ signaling, which favors the use of the conservative pathways NHEJ and HR over the error-prone ones (like MMEJ) [32]. Consequently, a more detailed cellular and molecular analysis is required to determine the context-dependent roles of DDR pathway choices and the presented ZEB1-Neon reporter may help to resolve these intricate findings.

## Conclusions

We established a very useful tool that allows following Zeb1 protein dynamics in individual cells by time-lapse imaging in tumor cells in culture as well as within the tumor by imaging PCTS cultures. These models will help to better understand how EMT is activated in specific regions of the tumor, how high and low Zeb1 levels impact cell phenotypes on an individual cell level, and how changes in metabolism, hypoxia, ROS levels, and secreted factors from the microenvironment affect ZEB1 expression. With the example of ZEB1, we demonstrate that detection of fluorescent protein tags by endogenous labeling is suitable even for genes that are expressed at low levels, like transcription factors. This versatile approach of live detection of dynamically regulated and moderately expressed proteins is likely applicable for other transcription factors and several other genes and opens new avenues for a much more detailed understanding of protein function.

## Methods

### Mouse strains, husbandry, and generation of Zeb1-Neon and KPC;Zeb1-Neon mice

Animal husbandry and experiments were approved by the state of Bavaria and performed according to the European Animal Welfare laws and guidelines. Mice were kept on a 12-h light–dark cycle in individually ventilated cages at a constant temperature between 20 and 24 °C and 45–65% humidity and provided with food and water ad libitum in the animal facilities of the Friedrich-Alexander University of Erlangen-Nürnberg. Euthanasia was performed using cervical dislocation or perfusion with PBS under anesthesia. Anesthesia procedure was performed using 2–5% isoflurane and analgesia provided with butorphanol (5 mg per kg bodyweight).

KPC mice have been described elsewhere [6, 33] and kept on a C57BL/6N background. To generate Zeb1-Neon mice, a synthetic single-stranded DNA megamer was designed and purchased (Integrated DNA Technologies, Inc.), containing 168-bp 5′-homologous sequences upstream and 274-bp 3′-homologous sequences downstream of the murine *Zeb1* stop codon, flanking a GGSGGSGGGS peptide linker sequence fused to the mNeonGreen coding sequence. To isolate fertilized oocytes, C57BL/6N mice were superovulated at an age of 26–30 days by i.p. injection of 5U PMSG 72 h and 5 U hCG 22 h before zygote retrieval. After injection of hCG, females were mated with C57BL/6N males and zygotes were flushed out from oviducts. Pronuclei of 410 zygotes were microinjected with a mix containing 40 ng/μl recombinant Cas9 (Integrated DNA Technologies, Inc.), 0.33 pmol/μl Alt-R® CRISPR-Cas9 tracrRNA duplex (protospacer AGAGAAGACAAATGAAGCTT, Integrated DNA Technologies, Inc.), and 60 ng/μl of the single-stranded HDR template (ssDNA). After microinjection into the pronucleus, 276 embryos developed to the two-cell stage overnight and were transferred into 16 pseudopregnant CD-1 foster mice. Twenty mice were born from which 11 showed correct homologous recombination events at 5′- (fwd: 5′-atcaccgctactcctactgc-3′; rev: 5′-atccggagccatctaccatg-3′; 780 bp) and 3′-ends (fwd: 5′-tccaagaccgagctcaactt; rev: 5′-gactgggtagcagcaggtag-3′; 585 bp) by PCR (55% efficiency). Three out of 11 founder mice were analyzed in detail and correct and seamless DNA integration was confirmed by Sanger sequencing. Offspring of heterozygous intercrosses were genotyped by PCR (fwd: 5′-aggaagtggaagcggatgaa-3′; rev: 5′-tgagcaggaaccatagcaca-3′) providing 280-bp wildtype and 1069-bp Zeb1-Neon knock-in bands. Embryos were isolated from timed matings by detection of vaginal plugs for staging.

KPC and Zeb1-Neon mice were mated to generate KPC;Zeb1-Neon tumors with heterozygous (ki/+) and homozygous (ki/ki) Zeb1-Neon knock-in genotypes. Tumor growth was monitored by palpation and mice were sacrificed at humane endpoints when tumors reached 1 cm^3^ volume for tumor isolation.

### Cell lines and culturing, TGFβ treatment, and siRNA transfection

MDA-MB-231 and MCF10A were purchased from American Type Culture Collection (ATCC) and cultured in DMEM (Gibco, 31966021)/10% FBS (Gibco, 10500064) and DMEM/F-12 (Gibco, 31331028)/5% horse serum (Gibco, 16050122)/20 ng/ml EGF (Peprotech, 100–15)/0.5 mg/ml hydrocortisone (Sigma, H0888)/0.1 mg/ml cholera toxin (Sigma, C8052)/10 mg/ml insulin (I9278), respectively, at 37 °C/5% CO_2_ in a humidified incubator as described previously [8]. Mouse embryonic fibroblasts (MEFs) and KPC;Zeb1-Neon tumor cells were isolated from E13.5 embryos and pancreatic tumors, respectively, as described previously [6, 31] and cultured similar to KPC661 in DMEM/10%FBS. H279 displayed a mesenchymal phenotype, while G623, G973, and G975 were more epithelial. Induction of EMT in MCF10A and KPC-derived cell lines was carried out by adding 5 ng/ml TGFβ1 (Peprotech, 100–21) with daily replacement of media and passaging every 2–3 days. During live cell imaging in the Incucyte S3 instrument, medium was replaced after 2 days.

For siRNA-mediated *ZEB1* knockdown, Silencer select siRNAs (Ambion; s229971 for si*ZEB1*, 4390844 for siCtrl) at a final concentration of 50 nM were transfected with Lipofectamine RNAiMAX transfection reagent (Thermo Fisher, 13778) according to the manufacturer’s instructions.

### Plasmids and cloning

pX330 (#42230) [23], pCRISPaint-mNeon-PuroR (#174090), and pCAS9-mCherry empty (#80975) [25] were purchased from Addgene. To generate a specific dual cut mNeonGreen donor plasmid for scarless fusion to ZEB1, an oligonucleotide (5′-cgggccagtacccaaaaaggtaggcgagagtagtgagcaagtgtctgaagaaaagacaaatgaagccgggtctggtggcagtggagggg-3′) that contained coding sequences for the C-terminal 17 amino acids of ZEB1 was inserted into the *Nhe*I/*Bam*HI site of pCRISPaint-mNeon-2A-puro. Subsequently, a second target sequence for the frame selector-derived sgRNA including the PAM site (5′-GGGCCAGTACCCAAAAAGGTagg-3′) was cloned into the *Apa*I/*Sal*I sites downstream of the puro sequences. The corresponding sgRNA attached to appropriate linkers was cloned into the *Bbs*I site of pX330 [23] for U6-mediated expression as frame selector. Likewise, the sgRNA sequence to target *ZEB1* in exon 9, most proximal to the stop codon (5′-agcagcttaggacaaaaagt-3′) and attached to appropriate linkers was cloned into the *Bbs*I site of pX330 to generate the target selector.

### CRISPaint-mediated ZEB1 tagging

Target selector pX330-ZEB1-Ex8 sgRNA, frame selector pX330-CRISPaint (Zeb1-Neon), Donor pCRISPaint-mNeon-2A-puro_ZEB1_scarless-dual-cut, and pCAS9-mCherry empty were transfected into MDA-MB-231 and MCF10A cells in ratios of 1:1:2:0 and 3:3:6:1, respectively, using FuGeneHD (Promega, E2311) according to the manufacturer’s instructions. Neon+ cells of MDA-MB-231 cells were isolated by FACS upon selection with 0.5 µg/ml puromycin for 2 days and clonally expanded in 96-wells. Thirty-eight (out of 94) were successfully expanded and tested by genotyping using primers for integration events at 5′- (fwd: 5′-cggacgagagagagagtttga-3′; rev: 5′-ggagaactggaggtcaccc-3′; 511 bp) and 3′-sites (fwd: 5′-tgcaagaactcttcctcacg-3′; rev: 5′-ctagaaaaacgattaggcttc-3′; 546 bp) of the targeted sequence. All 38 clones displayed correct 5′-integration events and out of 30 tested 5 displayed also correct 3′-integration. Correct sequence integrity was confirmed by Sanger sequencing, revealing an efficiency of 17%. mCherry+ MCF10A cells were clonally expanded in 96-wells 72 h after transfection. Nine out of 48 successfully expanded clones showed partial and 2 correct integration, which was confirmed by Sanger sequencing (4% efficiency).

### Immunoblotting and qRT-PCR

Protein was isolated upon cell lysis in RIPA buffer (150 mM NaCl, 50 mM Tris–HCl pH8.0, 0.5% Na-deoxycholate (w/v), 0.1% SDS (v/v), 1% NP-40 (v/v)), supplemented by 1 mM PMSF and 1 × complete protease inhibitor cocktail (Roche, 04693132001) on ice. Upon sedimentation of insoluble fractions by centrifugation, protein concentration was measured using BCA Protein Assay Kit (Thermo Fisher Scientific, 23225) following the manufacturer’s instructions and the FLUOstar Omega reader (BMG Labtech). After separation by 10% SDS-PAGE at 160 V, proteins were transferred to a nitrocellulose membrane (Roth, 4685.1) by wet blot transfer (Bio-Rad, 1620177) with Tris/glycine buffer for 2 h at 300 mA at 4 °C. Antibodies were diluted in 5% dry milk/TBS/0.1% Tween-20, incubated over night at 4 °C (primary) and 1 h at RT (secondary). For protein detection Western Lightning Plus ECL solution (Perkin-Elmer, NEL105001EA) or Clarity Western ECL Substrate (Bio-Rad, 1705061) was used and analyzed with the ChemiDoc MP Imaging System and ImageLab 6.1 software (Bio-Rad). Quantification was done with ImageJ v.1.54 and normalized to β-actin. The following antibodies were used: mouse anti-E-cadherin (BD Pharmingen, 610182; 1:5000), rabbit anti-EpCAM (abcam, ab32392, 1:1000), rabbit anti-fibronectin (abcam, ab2413, 1:5000), mouse anti-mNeonGreen (ChromoTek, 32F6, 1:1000), mouse anti-N-cadherin (BD Pharmingen, 610920, 1:2000), mouse anti-MRE11 (Novus, NB100-473, 1:1000), rabbit anti-NBS1 (Novus, NB100-143, 1:1000), mouse anti-RAD50 (GeneTex, GTX70228, 1:1000), mouse anti-αSMA (Sigma, A2547, 1:5000), rabbit anti-vimentin (Cell Signaling, 5741, 1:1000), rabbit anti-ZEB1 (Sigma Prestige, HPA027524; 1:2000), rabbit anti-ZEB1 (Proteintech, 21544-1-AP, 1:5000), mouse anti-β-actin (Sigma, A5441; 1:5000), rabbit anti-β-tubulin (Cell Signaling, 2146, 1:10,000), rabbit anti-GAPDH (Cell Signaling, 2118, 1:20,000), goat anti-mouse IgG HRP (Jackson ImmunoResearch, 115–035–146, 1:10,000), and goat anti-rabbit IgG HRP (Jackson ImmunoResearch, 111–035–144, 1:10,000).

Total RNA was isolated and reversely transcribed using the RNeasy Plus Mini kit (Qiagen, 74136) and the RevertAid First Strand cDNA Synthesis Kit (Thermo Fisher Scientific, K1622) as previously described [44]. cDNA was amplified in 384-well plates using gene-specific primers using the Universal Probe Library (Roche, 04869877001) and the TaqMan Universal Master Mix (Applied Biosystems, 4440040). Samples were run in triplicates in a LightCycler 480 II (Roche) and normalized to *ACTB*. Primer sequences and used UPLs: *ACTB* (fwd: 5′-ccaaccgcgagaagatga-3′; rev: 5′-ccagaggcgtacagggatag-3′; UPL#64), *CDH1* (fwd: 5′-ttgacgccgagagctacac-3′; rev: 5′-gtcgaccggtgcaatctt-3′; UPL#80), *CDH2* (fwd: 5′-tgcccaagacaaagagaccc-3′; rev: 5′-tggccactgtgcttactgaa-3′; UPL#124), *EPCAM* (fwd: 5′-atgccagtgtacttcagttggtgc-3′; rev: 5′-gccattcatttctgccttcatcacc-3′; UPL#71), *Neon* (fwd: 5′-tttggctccatcaacggtgt-3′; rev: 5′-aatccagggggagaactgga-3′; UPL#122), *VIM* (fwd: 5′-tggtctaacggtttccccta-3′; rev: 5′-gacctcggagcgagagtg-3′; UPL#56), *ZEB1* (fwd: 5′-aactgctgggaggatgacac-3′; rev: 5′-tcctgcttcatctgcctga-3′; UPL#57).

### Vibratome sectioning and PCTS

PCTSs were obtained from endpoint KPC;Zeb1-Neon mice using a Leica VT1200S vibratome. From freshly dissected tumors, a 6-mm core was excised with a biopsy punch (Kai medical, 48601) and embedded in low gelling agarose (Sigma, A9045). Two hundred-micrometer sections were generated with speed of 0.1 mm/s and amplitude of 2.5 mm. During sectioning, PCTSs were kept in PBS on ice. Slices were transferred to low-attachment 24-well plates (Thermo Fisher Scientific, 174930) and cultured in DMEM/F-12 (Gibco, 31331028)/10% FBS (Gibco, 10500064)/1% Penicillin-Streptomycin (Gibco, 15140122).

### Cryosectioning, immunofluorescence labeling, and imaging

For cryosectioning and fluorescence detection by intrinsic Neon fluorescence or immunolabeling, embryos were fixed for 2 h in 4% PFA/PBS, washed in PBS and incubated for 4 h/over night in 15%/30% sucrose/PBS before embedding into TissueTek OCT (Sakura Finetek, 4583), and sectioned at 10 µm. Frozen sections were thawed from −80 °C for 30 min at RT, fixed for 10 min in 4% PFA, permeabilized in PBS/0.25% Triton X100/100 mM glycine solution, and blocked for 30 min in blocking solution (3% BSA/PBS). Primary and secondary antibodies were diluted in blocking solution and incubated at 4 °C over night and at RT for 1 h, respectively, followed by 3 × washing steps in PBS, before final embedding in antifadent solution AF1 (Citifluor). To detect protein localization in cell lines, 1 × 10^5^ cells were plated on cover slips in 12-well plates and fixed 1 day later. Detection was similar to cryosections with 10-min fixation in 2% PFA/PBS and incubation times with primary and secondary antibodies for 1 h. After 30-min fixation in 4% PFA and 30-min permeabilization in 0.5% Triton X100/PBS, immunofluorescence on PCTSs was done by submerging slices in 500 µl antibody solution in µ-Plate 24-well imaging plates (ibidi, 82406), incubated overnight (primary) and 4 h (secondary) at 4 °C. Washing times were extended to 30 min each and slices mounted in antifadent solution AF1 (Citifluor). The following antibodies were used: rabbit anti-ZEB1 (Cell Signaling, 70512, 1:400), mouse anti-E-cadherin (BD Pharmingen, 610182; 1:200), mouse anti-mNeonGreen (ChromoTek, 32F6; 1:200), anti-mouse alexa488 (Thermo Fisher Scientific, A11029; 1:200), anti-rabbit alexa555 (Thermo Fisher Scientific, A21428; 1:200), anti-mouse alexa647 (Thermo Fisher Scientific, A21235; 1:200), and anti-mouse CF640R (Sigma, SAB4600343; 1:200). Imaging of cells and cryosections was done using a Leica DM5500 microscope and LAS X software. Immunofluorescence of PCTSs was imaged on a Spinning Disc Axio Observer Z1 microscope (Zeiss) attached to an EVOLVE 512 EMCCD camera (Ikona Imaging) with ZEN software (Zeiss).

MDA-MB-231 live cell time-lapse imaging was done by plating 2000 cells in µ-Slide 8-well glass bottom slide (ibidi, 80827) Spinning Disc Axio Observer Z1 microscope with image acquisition every 20 min. For regular live cell imaging to detect Neon fluorescence, medium was replaced by FluroBrite DMEM (Gibco, A1896701), supplemented with 10% FBS and GlutaMAX (Gibco, 35050061), and imaged at EVOS M7000 (Thermo Fisher Scientific) and DM IL LED standard cell culture microscope (Leica). Fresh or cultured PCTSs were imaged in DMEM/F-12 (Gibco, 31331028)/10% FBS (Gibco, 10500064)/1% Penicillin-Streptomycin (Gibco, 15140122) at the EVOS M7000 System (Thermo Fisher Scientific).

For detection of Neon fluorescence in freshly dissected embryos, specimens were mounted in PBS in a glass Petri dish observed under an Axio Zoom.V16 microscope (Zeiss) and ZEN software.

All images were processed and time-lapse movies generated by using ImageJ v.1.54 and the 3Dscript plugin [45].

### Incucyte time-lapse imaging and data processing

For time-lapse imaging of KPC;Zeb1-Neon tumor cells, 1000 cells were plated into a regular flat-bottom plastic 96-well plate (TPP 92096). Cells were either kept untreated or treated with 5 ng/ml TGFβ1 for the indicated time with daily replacing the medium. After plating in Fluorobrite medium (Gibco, A1896701)/10% FBS/GlutaMAX (Gibco, 35050061), plates were kept for additional 16 h in a regular cell culture incubator before transfer into the Incucyte S3 (Sartorius) for imaging at 2-h intervals using Incucyte 2022B rev2 application suite (Sartorius) for acquisition and image analysis. Exported images were processed for time-lapse movies with ImageJ.

For quantification of the dynamic expression of Zeb1-Neon and the fractions of Zeb1hi cells, Incucyte-generated images were processed using CellProfiler 4 [46] for cell segmentation and densitometry of nuclear Zeb1-Neon intensities. Informed by our previous work [22], we defined Zeb1hi cells as the upper 10% of the intensity sum distribution, averaged across all cells not treated with TGFβ. Individual time-points in untreated and TGFβ-treated conditions were then assigned the corresponding fraction of Zeb1hi cells.

### Flow cytometry

Transfected MDA-MB-231 and MCF10A cells were trypsinized, washed in PBS, and resuspended in FACS buffer (PBS/2% FBS)/5 mM EDTA. Neon+ (MDA-MB-231) and mCherry+ (MCF10A) cells were sorted as single cells into wells of a 96-well plate using MoFlo XDP (Beckman Coulter). For detection of specific ZEB1-Neon fluorescence intensity, 6 × 10^5^ cells were transferred to FACS tubes and resuspended in 100 µl PBS, adding Zombie NIR (BioLegend, 77184; 1:500). After incubation for 15 min in the dark on ice, cells were washed and resuspended in 500 µl FACS buffer/5 mM EDTA for acquisition at a Cytoflex S with CytExpert software (Beckmann Coulter). Kaluza 2.2 was used for downstream analysis.

### Statistics

Data analysis was performed using GraphPad Prism 10.5 software. For multiple comparisons, one-way ANOVA with Dunnett’s post hoc test was applied. *p* values < 0.05 were considered statistically significant. Sample size and replicates are indicated in the figure legends. All experiments presented were repeated in at least three independent biological replicates. Data are presented as mean ± S.E.M. of *n* observations, where *n* represents the individual number of experiments. Data distribution was assumed to be normal but not formally tested. *p < 0.05; **p < 0.01; ***p < 0.001; ****p < 0.0001.

## Supplementary Information

Additional file 1.

Additional file 2.

Additional file 3.

Additional file 4.

## Data Availability

All data generated or analyzed during this study are included in this published article and its supplementary information files. The datasets used and analyzed during the current study are available from the corresponding author on reasonable request.

## Abbreviations

BF: Bright-field
DDR: DNA damage response
DSB: Double-strand break
EMT: Epithelial-mesenchymal transition
HR: Homologous recombination
IF: Immunofluorescence staining
MEFs: Mouse embryonic fibroblasts
MET: Mesenchymal-epithelial transition
MFI: Mean fluorescence intensity
MMEJ: Microhomology-mediated end joining
Neon: mNeonGreen (monomeric NeonGreen fluorescent protein)
NHEJ: Non-homologous end joining
PCTS/PCTSs: Precision-cut tissue slice/slices
PDAC: Pancreatic ductal adenocarcinoma
ROS: Reactive oxygen species
ssDNA: Single-stranded DNA
ZEB1hi: Cells with high levels of ZEB1
ZEB1lo: Cells with low levels of ZEB1
