# Supplementary material for: A ZEB1-Neon knock-in uncovers traceable dynamics of epithelial-mesenchymal transition in tumors in vivo
Source: BMC Biol. 2026 May 12;24:116. doi: 10.1186/s12915-026-02629-0 (PMC13170155; doi:10.1186/s12915-026-02629-0)
Supplement: Supplementary file 1 — Additional file 1. [file 12915_2026_2629_MOESM1_ESM.pdf]

A ZEB1-Neon knock-in uncovers traceable dynamics of epithelial-mesenchymal transition in tumors *in vivo*

Elisabetta D'Avanzo[1], Amelie Mahr[1], Nicolas Sauer[1], Simon Brandt[1], Ruthger van Roey[1], Harald Schuhwerk[1,2], Philipp Tripal[3], Benjamin Schmid[3], Stefanie Brey[4], Thomas H. Winkler[4], Simone Brabletz[1,5], Thomas Brabletz[1,5], and Marc P. Stemmler[1,*]

1 Department of Experimental Medicine 1, Nikolaus-Fiebiger Center for Molecular Medicine, Friedrich-Alexander University of Erlangen-Nürnberg (FAU), Erlangen, Germany.
2 Department of Dermatology, University Hospital Regensburg, Regensburg, Germany
3 Optical Imaging Competence Centre Erlangen (OICE), Friedrich-Alexander University of Erlangen-Nürnberg (FAU), Erlangen, Germany.
4 Division of Genetics, Department of Biology, Friedrich-Alexander University of Erlangen-Nürnberg (FAU), Erlangen, Germany
5 Comprehensive Cancer Center Erlangen-EMN (CCC ER-EMN), Bavarian Cancer Research Center (BZKF), Erlangen, Germany

*correspondence to: Marc Stemmler

Experimental Medicine 1
Nikolaus-Fiebiger Center for Molecular Medicine
Glückstr. 6
91054 Erlangen, Germany
Tel. ++49 761 85 29101
marc.stemmler@fau.de

Additional file 1: Fig. S1-S6.pdf.

Figure S1. Confirmation of successful sequence integration by CRISPaint to generate ZEB1-Neon tagging in Exon 9 of the ZEB1 locus in MDA-MB-231 cells.

Figure S2. Functional evaluation of the ZEB1-Neon allele in MCF10A cells.

Figure S3. Confirmation of successful generation of a Zeb1-Neon mouse line by targeting Exon 8 of the Zeb1 locus by CRISPR/Cas9-mediated homologous recombination.

Figure S4. Analysis of ZEB1hi and ZEB1lo MDA-MB-231 cells.

Figure S5. Protein analysis of TGFβ treated KPC;Zeb1-Neon PDAC cell lines.

Figure S6. In vivo detection of Zeb1-Neon dynamics in KPC;Zeb1-Neon PDAC cell lines.

Suppl. Fig. S1

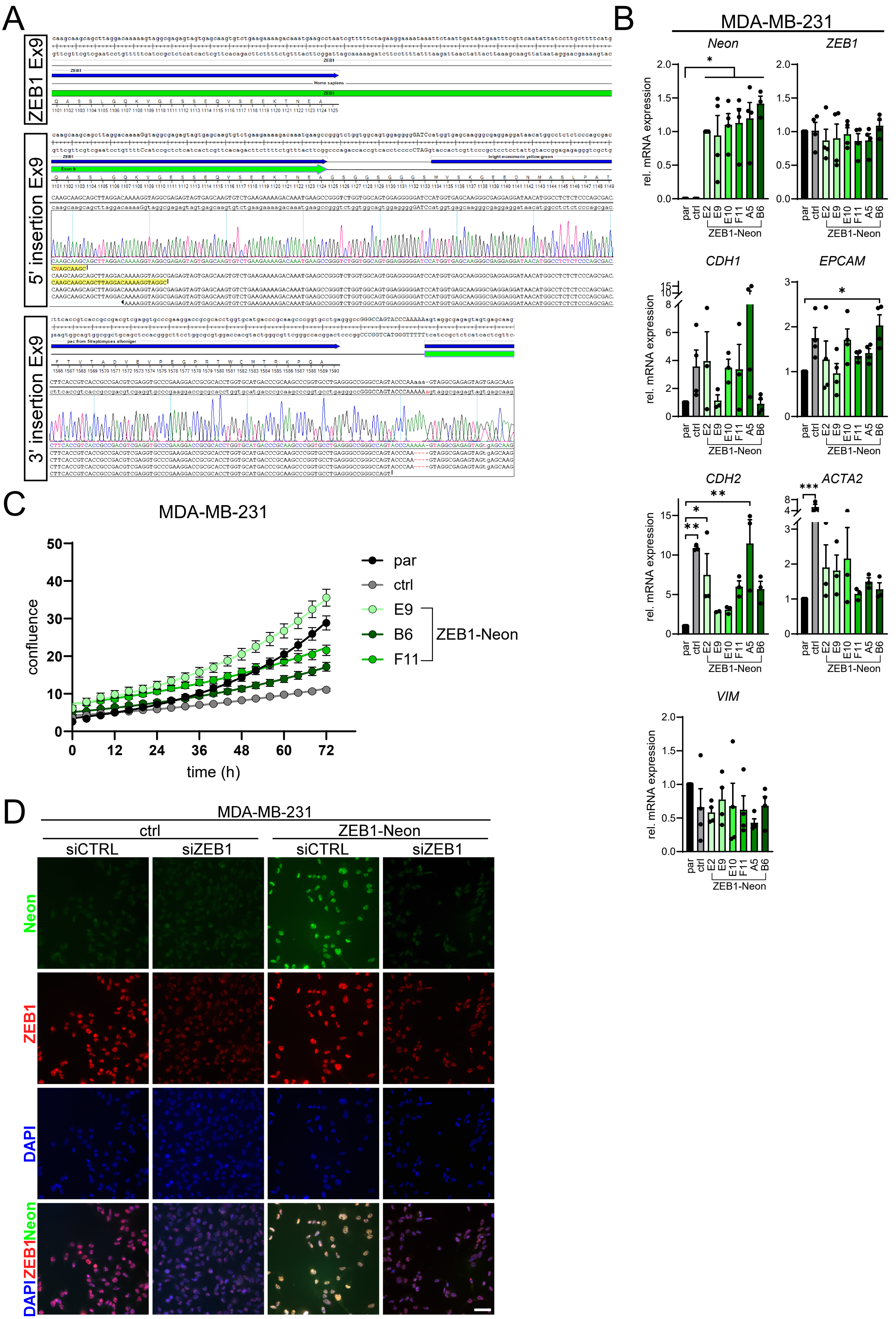
A
ZEB1 Ex9
5' insertion Ex9
3' insertion Ex9
B
MDA-MB-231
Neon
ZEB1
CDH1
EPCAM
CDH2
ACTA2
VIM
rel. mRNA expression
par
ctrl
E2
E9
E10
F11
A5
B6
ZEB1-Neon
C
MDA-MB-231
confluence
time (h)
par
ctrl
E9
B6
F11
ZEB1-Neon
D
MDA-MB-231
ctrl
ZEB1-Neon
siCTRL
siZEB1
Neon
ZEB1
DAPI
DAPIZEB1Neon

Figure S1. Confirmation of successful sequence integration by CRISPaint to generate ZEB1-Neon tagging in Exon 9 of the ZEB1 locus in MDA-MB-231 cells. (A) Alignment of *in silico* assembled sequences and experimental chromatograms of the wildtype *ZEB1* locus and ZEB1-Neon insertion events at 5'- and 3'-regions of *ZEB1* Exon 9, confirming proper integration and scarless fusion. Note, that the majority of clones show a small presumably irrelevant deletion in a polyA-stretch at the 3'-end outside of the coding sequence. (B) qRT-PCR analysis of several EMT marker genes, confirming robust ZEB1-Neon and no decrease in overall ZEB1 expression as well as only small changes in the EMT continuum. Note, that fold-changes in *CDH1* and *EPCAM* are observed at very low expression levels and fluctuations are not dramatically changing protein levels (n=3 experiments). (C) Incucyte live-cell imaging demonstrates small clonal variability in proliferation, independent of ZEB1-Neon integration (n=1, 10 replicates). (D) IF images of MDA-MB-231 ctrl and E9 ZEB1-Neon clones to demonstrate loss of Neon immunodetection along with *ZEB1* loss upon si*ZEB1* mediated knockdown and specificity of the ZEB1-Neon signal. Nuclei are stained with DAPI. Scale bar, 50 µm.

Suppl. Fig. S2

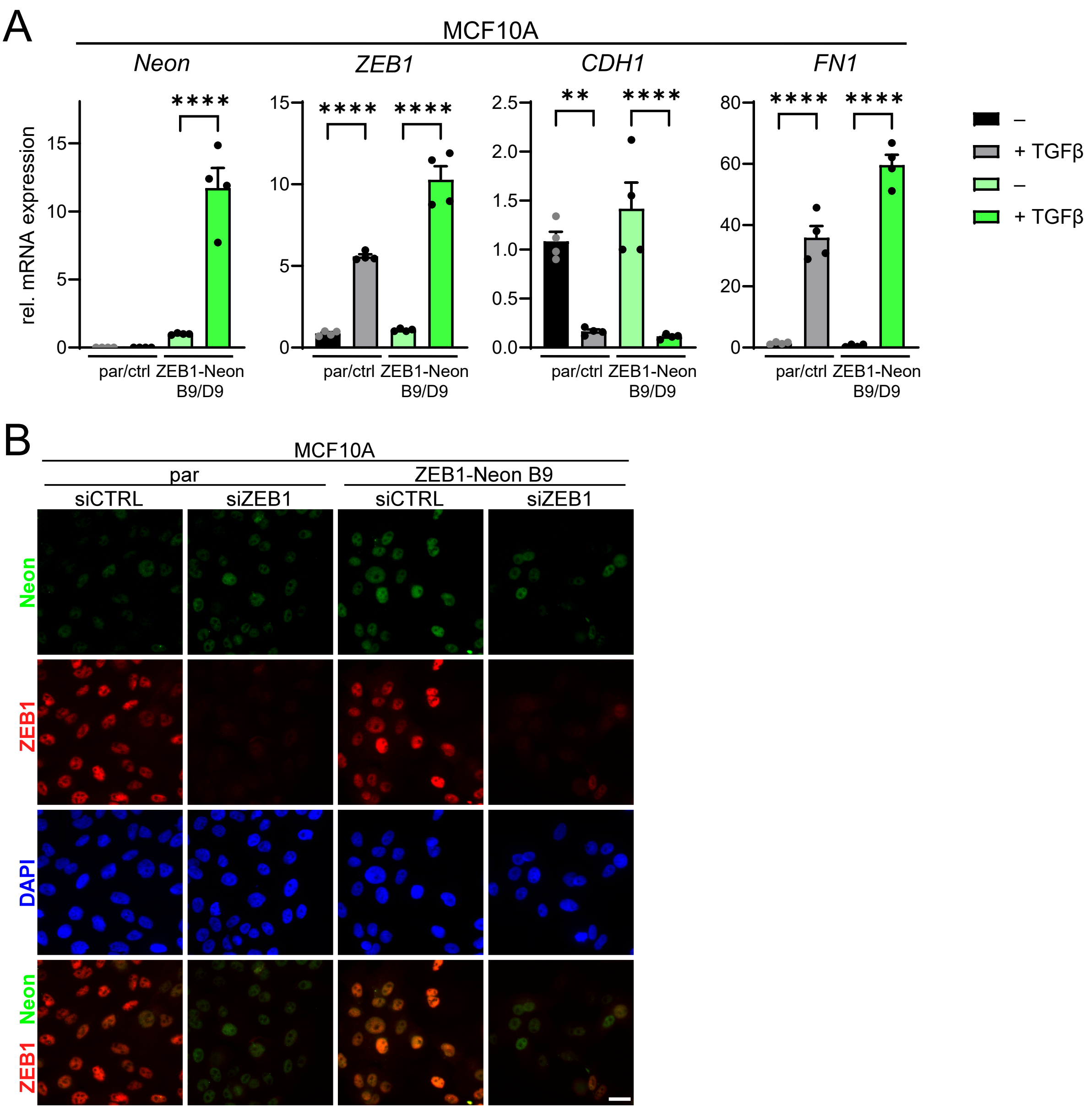
A
MCF10A
Neon
ZEB1
CDH1
FN1
rel. mRNA expression
****
**
par/ctrl
ZEB1-Neon B9/D9
–
+ TGFβ
–
+ TGFβ
B
MCF10A
par
ZEB1-Neon B9
siCTRL
siZEB1
siCTRL
siZEB1
Neon
ZEB1
DAPI
Neon
ZEB1

Figure S2. Functional evaluation of the ZEB1-Neon allele in MCF10A cells. (A) qRT-PCR analysis of *ZEB1*, *Neon*, *CDH1* and *FN1* expression upon 14d TGFβ treatment to confirm (*ZEB1-*)*Neon* transcript activation together with *ZEB1* during EMT induction. Par and ctrl and ZEB1-Neon B9 and D9 cells were combined (n=4; 2 experiments for each cell line) (E) IF images of MCF10A F8 ctrl and B9 ZEB1-Neon clones treated with TGFβ and siRNA transfection as in (D) to demonstrate loss of Neon immunodetection along with *ZEB1* loss and specificity of the ZEB1-Neon signal. Nuclei are stained with DAPI. Scale bar, 25 µm.

Suppl. Fig. S3

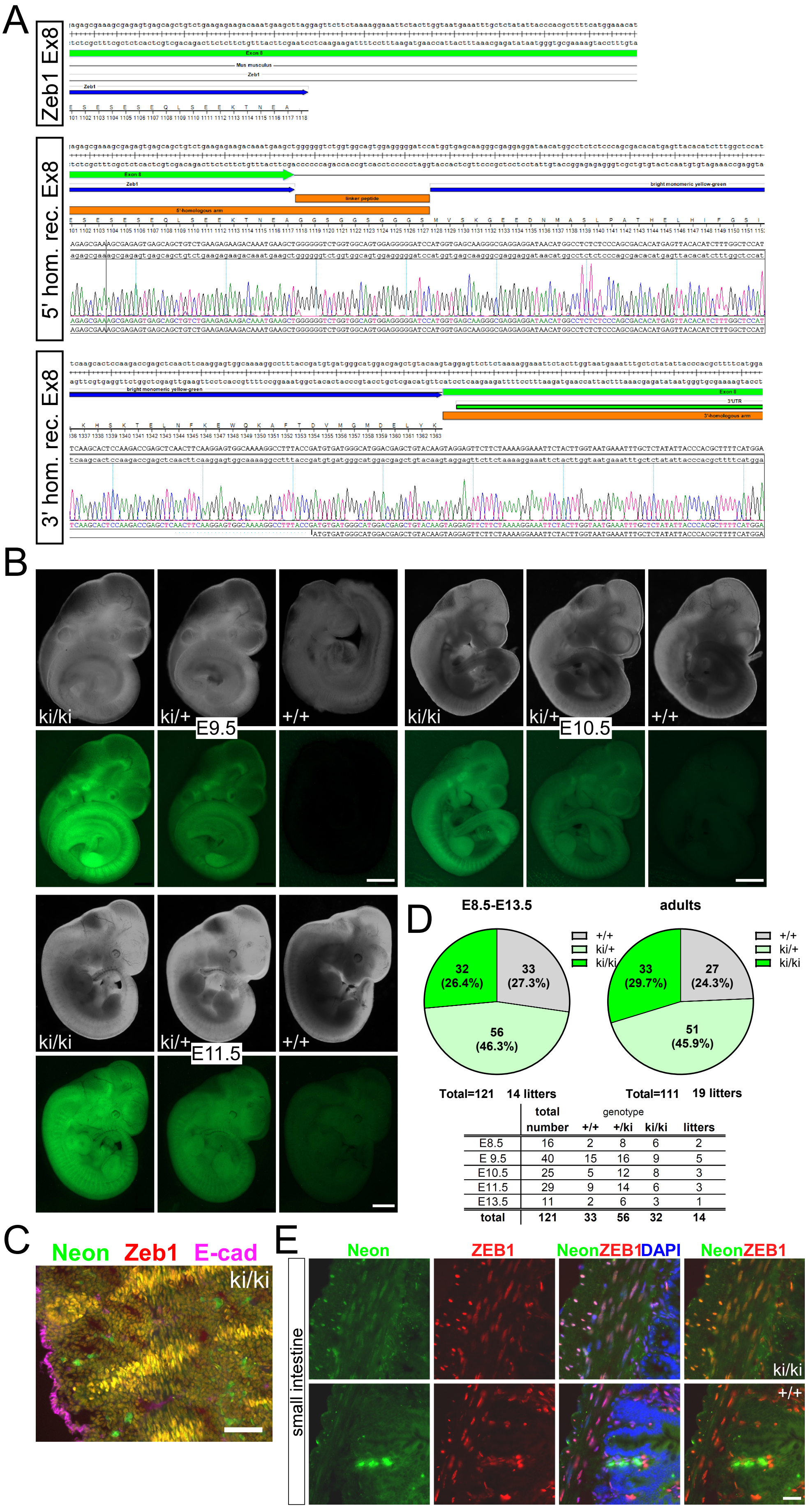

	total number	genotype +/+	+/ki	ki/ki	litters
E8.5	16	2	8	6	2
E 9.5	40	15	16	9	5
E10.5	25	5	12	8	3
E11.5	29	9	14	6	3
E13.5	11	2	6	3	1
**total**	**121**	**33**	**56**	**32**	**14**

Figure S3. Confirmation of successful generation of a Zeb1-Neon mouse line by targeting Exon 8 of the Zeb1 locus by CRISPR/Cas9-mediated homologous recombination. (A) Alignment of sequences and experimental chromatograms of the wildtype *Zeb1* locus and successful Zeb1-Neon insertion events at 5'- and 3'-regions of Exon 8, confirming proper integration and sequence integrity. (B) Fluorescence imaging of whole-mount Zeb1-Neon ki/ki, ki/+ and +/+ embryos between E9.5 and E11.5, demonstrating a gene-dosage dependent increase in fluorescence intensity from Zeb1-Neon nuclear protein. (C) Sagittal section of a cryoembedded Zeb1-Neon ki/ki embryo at E11.5 and IF labeling of Zeb1 (red) and E-cad (magenta), showing increased Neon fluorescence in dorsal root ganglia with a perfect overlap of anti-ZEB1 detection. (D) Genotype distribution of offspring from heterozygous intercrosses, between E8.5 and E13.5 and in adults, showing normal mendelian distribution. (E) Zeb1-Neon fluorescence is also detected in adult cryoembedded tissue sections like muscle layers and the lamina propria of the small intestine. Nuclei are stained with DAPI. Scale bars, 500 µm (E9.5), 1 mm (E10.5 and E11.5), 50 µm (C) and 25 µm (E).

Suppl. Fig. S4

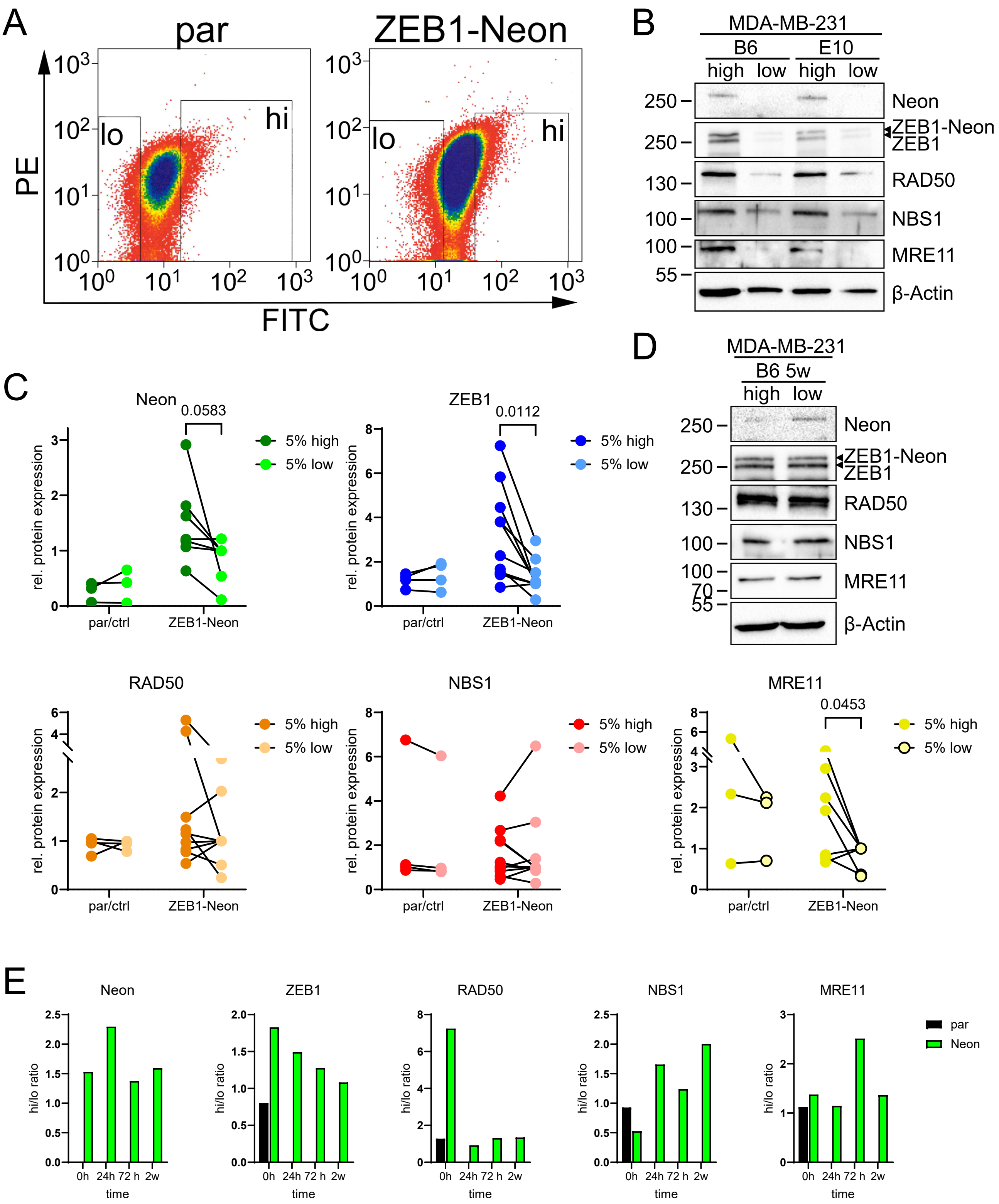
A
par
ZEB1-Neon
PE
FITC
lo
hi
B
MDA-MB-231
B6
E10
high
low
high
low
250
250
130
100
100
55
Neon
ZEB1-Neon
ZEB1
RAD50
NBS1
MRE11
β-Actin
C
Neon
0.0583
5% high
5% low
rel. protein expression
par/ctrl
ZEB1-Neon
ZEB1
0.0112
RAD50
NBS1
MRE11
0.0453
D
MDA-MB-231
B6 5w
high
low
250
250
130
100
100
70
55
Neon
ZEB1-Neon
ZEB1
RAD50
NBS1
MRE11
β-Actin
E
Neon
ZEB1
RAD50
NBS1
MRE11
hi/lo ratio
0h
24h
72 h
2w
time
par
Neon

Figure S4. Analysis of ZEB1hi and ZEB1lo MDA-MB-231 cells. (A) Representative FACS plot of isolating ZEB1 high and low populations in the FITC (Neon) channel together with the distribution in an unrelated channel (PE). Note, that the whole population between par and ZEB1-Neon cells robustly shifts from background to higher intensities. (B) Western blot of two clones sorted for ZEB1-Neon hi and lo cells, analyzed directly after sorting. (C) Quantification of Western blot repetitions from (B) and Fig. 4A, combined in par/ctrl (one each) and Zeb1-Neon clones E9, B6 and A5 normalized to β-Actin, demonstrating a robust separation into ZEB1hi and ZEB1lo cells that is correlated with RAD50 and MRE11, whereas NBS1 is more variable (n=3-8 experiments). (D) ZEB1hi and ZEB1lo sorted cells analyzed by Western blot after 5 weeks (5w) culturing, demonstrating the transient nature of ZEB1hi and ZEB1lo states. (E) Quantification of Western blot from Fig. 4B normalized to β-Actin showing the dynamic changes of protein expression between ZEB1hi and ZEB1lo cells only in ZEB1-Neon cells (Neon).

Suppl. Fig. S5

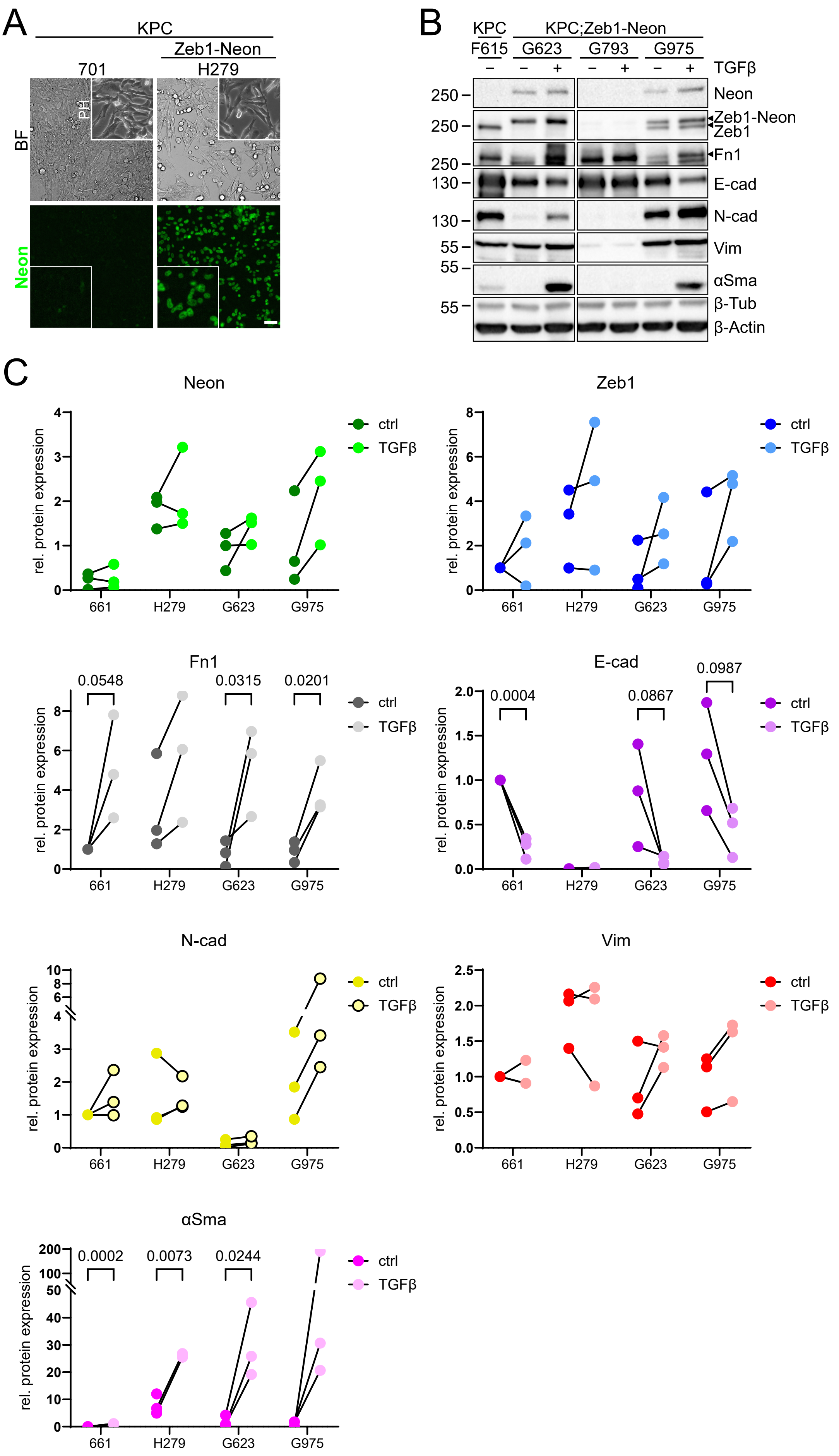
A
KPC
Zeb1-Neon
701
H279
BF
PH
Neon
B
KPC
KPC;Zeb1-Neon
F615
G623
G793
G975
TGFβ
250
130
55
Neon
Zeb1-Neon
Zeb1
Fn1
E-cad
N-cad
Vim
αSma
β-Tub
β-Actin
C
Neon
Zeb1
Fn1
E-cad
N-cad
Vim
αSma
rel. protein expression
ctrl
TGFβ
661
H279
G623
G975
0.0548
0.0315
0.0201
0.0004
0.0867
0.0987
0.0002
0.0073
0.0244

Figure S5. Protein analysis of TGFβ treated KPC;Zeb1-Neon PDAC cell lines. (A) Fluorescence *in vivo* imaging of mesenchymal cell lines KPC;Zeb1-Neon ki/ki (H279) and KPC701 ($Zeb1^{+/+}$) using the EVOS system. (B) Western blot analysis of epithelial KPC cells F615 (+/+) and epithelial KPC;Zeb1-Neon cells G623 (ki/ki), G793 (ki/ki) and G975 (ki/+) with and without 12d of TGFβ treatment. TGFβ-induced EMT after 12d show increased Zeb1(-Neon) expression accompanied by changes in EMT markers, except for G793 which has very low Zeb1 and Zeb1-Neon levels even after TGFβ exposure. (C) Quantification of Western blot repetitions from KPC;Zeb1-Neon PDAC cell lines treated for 8d with TGFβ in Fig. 4D normalized to β-Actin to demonstrate robust EMT induction by specific EMT markers, correlated with upregulation of Zeb1 and Zeb1-Neon (n=3 experiments). Scale bar, 50 µm.

Suppl. Fig. S6

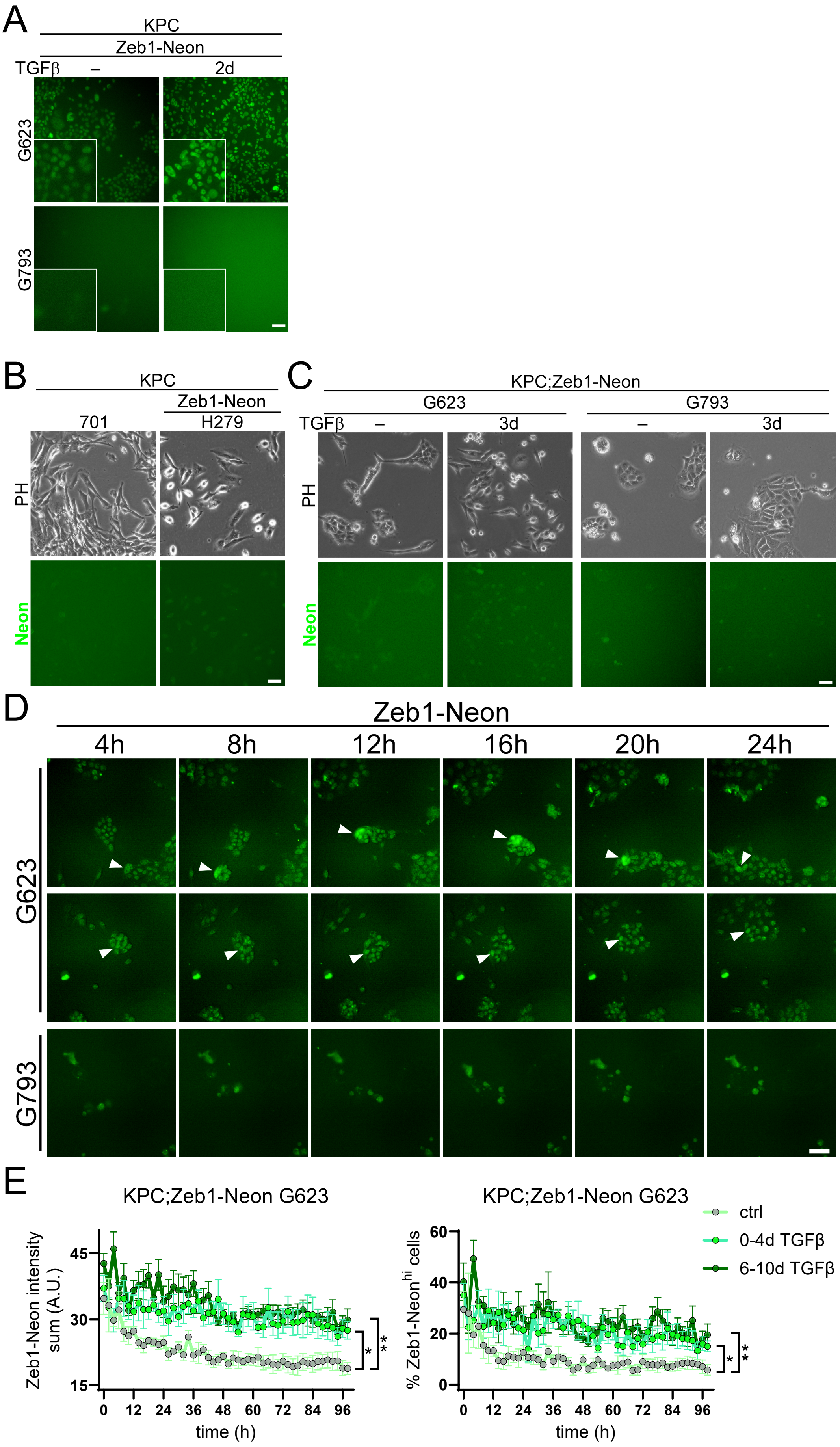
A
KPC
Zeb1-Neon
TGFβ
–
2d
G623
G793
B
KPC
Zeb1-Neon
701
H279
PH
Neon
C
KPC;Zeb1-Neon
G623
G793
TGFβ
–
3d
–
3d
PH
Neon
D
Zeb1-Neon
4h
8h
12h
16h
20h
24h
G623
G793
E
KPC;Zeb1-Neon G623
KPC;Zeb1-Neon G623
Zeb1-Neon intensity sum (A.U.)
% Zeb1-Neon^hi cells
time (h)
time (h)
ctrl
0-4d TGFβ
6-10d TGFβ
*
**
*
**

Figure S6. In vivo detection of Zeb1-Neon dynamics in KPC;Zeb1-Neon PDAC cell lines. (A) Fluorescence *in vivo* imaging of two untreated epithelial KPC;Zeb1-Neon ki/ki cell lines (G623 and G793) and after 2d TGFβ treatment using the EVOS system. Note, that despite of the Zeb1-Neon ki/ki genotype in G793 cells, no Neon-specific fluorescence was detected, paralleling Zeb1 absence. (B,C) Standard inverted microscopy of cultured KPC and KPC;Zeb1-Neon cells, demonstrating that specific Neon expression is detected in mesenchymal cells (H279) and increases after 3d TGFβ treatment of epithelial cells (G623), whereas no specific signals are detected in KPC701 (+/+) and the Zeb1-negative line G793. (D) Still images of 4d time-lapse movies of G623 (upper/middle) and G793 cells (lower panel) cultured in the Incucyte to detect Zeb1-Neon fluorescence (Suppl. Movies 4-6). Zeb1hi cells/clusters are marked by a white arrowhead, indicating maintenance of Zeb1hi status over time that display a specific high mobility phenotype (upper panel). Interesting intercepts were extracted and starting points set to 4h. (E) Quantification of sum intensity (left) and percent Zeb1hi cells based on Zeb1-Neon nuclear expression during time-lapse imaging of KPC;Zeb1-Neon G623 cells (n=4-6) to demonstrate that Zeb1 and Zeb1hi percentages are increasing during TGFβ exposure, mainly during the first day of treatment (0-4d) and stay relatively constant afterwards and during 6-10d TGFβ treatment. Multiple wells and images from Incucyte live-cell imaging were analyzed by CellProfiler. Note, that untreated (ctrl) cells reduce fluorescence sum intensity and the fraction of Zeb1hi cells, indicating that the kinetic changes of Zeb1 expression and Neon detection are more complex and need further investigation that also covers potential changes in intensity induced by cell shape and photobleaching. Scale bars, 50 µm.

Supplementary Video description

Video S1. Confocal fluorescence time-lapse imaging of MDA-MB-231 ctrl and E2 clones for 7 hours, imaged every 10 min in DIC and green channel overlay; 100 µm x 100 µm.

Video S2. Fluorescence live cell imaging of freshly isolated and sectioned PCTS of a KPC;Zeb1 ki/+ tumor, focusing through 2 areas to visualize nuclear staining of Zeb1-Neon positive cells within the tumor slice; 635 µm x 476 µm (EVOS sytem).

Video S3. 3D reconstruction of a confocal z-stack of the KPC;Zeb1-Neon ki/+ tumor PCTS from Fig. 4H; 100 µm x 100 µm.

Video S4. Fluorescence live cell imaging of (Neon-positive) G623 KPC;Zeb1-Neon ki/ki cells (Fig. 4I, upper panel) with green (left) and BF/green channel overlay (right) for 4 days, imaged every 2 hours (Incucyte S3). A very bright Zeb1-Neon positive cluster is highlighted by arrowheads. Scale (time-stamp box), 100 µm.

Video S5. Fluorescence live cell imaging of (Neon-positive) G623 KPC;Zeb1-Neon ki/ki cells (Fig. 4I, middle panel) with green (left) and BF/green channel overlay (right) for 4 days, imaged every 2 hours (Incucyte S3). A bright Zeb1-Neon positive cell is highlighted by arrowheads. Scale (time-stamp box), 100 µm.

Video S6. Fluorescence live cell imaging of (Neon-negative) G793 KPC;Zeb1-Neon ki/ki cells (Fig. 4I, lower panel) with green (left) and BF/green channel overlay (right) for 4 days, imaged every 2 hours (Incucyte S3). Note, that only dead cells/debris show autofluorescence. Scale (time-stamp box), 100 µm.

Video S7. Composite video from individual time-lapse recordings of live cell imaging of untreated KPC661 cells (left) and upon EMT induction by TGFβ treatment for 0-4 (middle) and 6-10 days. Bright-field and green channels are shown as overlay (upper) and green fluorescence channel alone (lower panels) imaged every 2 hours (Incucyte S3), showing only background fluorescence (see also Fig. 5A). Scale (time-stamp box), 100 µm.

Video S8. Composite video from individual time-lapse recordings of live cell imaging of untreated mesenchymal KPC;Zeb1-Neon H279 ki/ki cells (left) and upon EMT induction by TGFβ treatment for 0-3 (middle) and 6-10 days. Bright-field and green channels are shown as overlay (upper) and green fluorescence channel alone (lower panels) imaged every 2 hours (Incucyte S3), showing high levels of Neon fluorescence that are not changing during TGFβ treatment (see also Fig. 5A,C). Scale (time-stamp box), 100 µm.

Video S9. Composite video from individual time-lapse recordings of live cell imaging of untreated epithelial KPC;Zeb1-Neon G623 ki/ki cells (left) and upon EMT induction by TGFβ treatment for 0-4 (middle) and 6-10 days. Bright-field and green channels are shown as overlay (upper) and green fluorescence channel alone (lower panels) imaged every 2 hours (Incucyte S3), showing moderate levels of Neon fluorescence that are increasing during 0-4 d TGFβ treatment (see also Fig. 5A,B). Scale (time-stamp box), 100 µm.

Video S10. Composite video from individual time-lapse recordings of live cell imaging of untreated epithelial KPC;Zeb1-Neon G975 ki/+ cells (left) and upon EMT induction by TGFβ treatment for 0-4 (middle) and 6-10 days. Bright-field and green channels are shown as overlay (upper) and green fluorescence channel alone (lower panels) imaged every 2 hours (Incucyte S3), showing low levels of Neon fluorescence that are increasing during 0-4 d TGFβ treatment (see also Fig. 5A). Scale (time-stamp box), 100 µm.
