# Supplementary material for: A ZEB1-Neon knock-in uncovers traceable dynamics of epithelial-mesenchymal transition in tumors in vivo
Source: BMC Biol. 2026 May 12;24:116. doi: 10.1186/s12915-026-02629-0 (PMC13170155; doi:10.1186/s12915-026-02629-0)
Supplement: Supplementary file 2 — Additional file 2. [file 12915_2026_2629_MOESM2_ESM.zip › Additional file 2, Videos S1-S10 file description.pdf]

A ZEB1-Neon knock-in uncovers traceable dynamics of epithelial-mesenchymal transition in tumors *in vivo*

Elisabetta D'Avanzo[1], Amelie Mahr[1], Nicolas Sauer[1], Simon Brandt[1], Ruthger van Roey[1], Harald Schuhwerk[1,2], Philipp Tripal[3], Benjamin Schmid[3], Stefanie Brey[4], Thomas H. Winkler[4], Simone Brabletz[1,5], Thomas Brabletz[1,5], and Marc P. Stemmler[1,*]

1 Department of Experimental Medicine 1, Nikolaus-Fiebiger Center for Molecular Medicine, Friedrich-Alexander University of Erlangen-Nürnberg (FAU), Erlangen, Germany.
2 Department of Dermatology, University Hospital Regensburg, Regensburg, Germany
3 Optical Imaging Competence Centre Erlangen (OICE), Friedrich-Alexander University of Erlangen-Nürnberg (FAU), Erlangen, Germany.
4 Division of Genetics, Department of Biology, Friedrich-Alexander University of Erlangen-Nürnberg (FAU), Erlangen, Germany
5 Comprehensive Cancer Center Erlangen-EMN (CCC ER-EMN), Bavarian Cancer Research Center (BZKF), Erlangen, Germany

*correspondence to: Marc Stemmler

Experimental Medicine 1
Nikolaus-Fiebiger Center for Molecular Medicine
Glückstr. 6
91054 Erlangen, Germany
Tel. ++49 761 85 29101
marc.stemmler@fau.de

Additional file 2: Videos S1-S10.zip.

Video S1. Confocal fluorescence time-lapse imaging of MDA-MB-231 ctrl and E2 clones for 7 hours.

Video S2: Fluorescence live cell imaging of freshly isolated and sectioned PCTS of a KPC;Zeb1 ki/+ tumor.

Video S3: 3D reconstruction of a confocal z-stack of the KPC;Zeb1-Neon ki/+ tumor PCTS.

Video S4: Fluorescence live cell imaging of (Neon-positive) G623 KPC;Zeb1-Neon ki/ki cells.

VideoS5: Fluorescence live cell imaging of (Neon-positive) G623 KPC;Zeb1-Neon ki/ki cells.

Video S6: Fluorescence live cell imaging of (Neon-negative) G793 KPC;Zeb1-Neon ki/ki cells.

Video S7: Composite video from individual time-lapse recordings of live cell imaging of untreated KPC661 cells (left) and upon EMT induction by TGFβ treatment for 0-4 (middle) and 6-10 days.

Video S8: Composite video from individual time-lapse recordings of live cell imaging of untreated mesenchymal KPC;Zeb1-Neon H279 ki/ki cells (left) and upon EMT induction by TGFβ treatment for 0-3 (middle) and 6-10 days.

Video S9: Composite video from individual time-lapse recordings of live cell imaging of untreated epithelial KPC;Zeb1-Neon G623 ki/ki cells (left) and upon EMT induction by TGFβ treatment for 0-4 (middle) and 6-10 days.

Video S10: Composite video from individual time-lapse recordings of live cell imaging of untreated epithelial KPC;Zeb1-Neon G975 ki/+ cells (left) and upon EMT induction by TGFβ treatment for 0-4 (middle) and 6-10 days.

Video S1. Confocal fluorescence time-lapse imaging of MDA-MB-231 ctrl and E2 clones for 7 hours, imaged every 10 min in DIC and green channel overlay; 100 µm x 100 µm.

Video S2. Fluorescence live cell imaging of freshly isolated and sectioned PCTS of a KPC;Zeb1 ki/+ tumor, focusing through 2 areas to visualize nuclear staining of Zeb1-Neon positive cells within the tumor slice; 635 µm x 476 µm (EVOS sytem).

Video S3. 3D reconstruction of a confocal z-stack of the KPC;Zeb1-Neon ki/+ tumor PCTS from Fig. 4H; 100 µm x 100 µm.

Video S4. Fluorescence live cell imaging of (Neon-positive) G623 KPC;Zeb1-Neon ki/ki cells (Fig. 4I, upper panel) with green (left) and BF/green channel overlay (right) for 4 days, imaged every 2 hours (Incucyte S3). A very bright Zeb1-Neon positive cluster is highlighted by arrowheads. Scale (time-stamp box), 100 µm.

Video S5. Fluorescence live cell imaging of (Neon-positive) G623 KPC;Zeb1-Neon ki/ki cells (Fig. 4I, middle panel) with green (left) and BF/green channel overlay (right) for 4 days, imaged every 2 hours (Incucyte S3). A bright Zeb1-Neon positive cell is highlighted by arrowheads. Scale (time-stamp box), 100 µm.

Video S6. Fluorescence live cell imaging of (Neon-negative) G793 KPC;Zeb1-Neon ki/ki cells (Fig. 4I, lower panel) with green (left) and BF/green channel overlay (right) for 4 days, imaged every 2 hours (Incucyte S3). Note, that only dead cells/debris show autofluorescence. Scale (time-stamp box), 100 µm.

Video S7. Composite video from individual time-lapse recordings of live cell imaging of untreated KPC661 cells (left) and upon EMT induction by TGFβ treatment for 0-4 (middle) and 6-10 days. Bright-field and green channels are shown as overlay (upper) and green fluorescence channel alone (lower panels) imaged every 2 hours (Incucyte S3), showing only background fluorescence (see also Fig. 5A). Scale (time-stamp box), 100 µm.

Video S8. Composite video from individual time-lapse recordings of live cell imaging of untreated mesenchymal KPC;Zeb1-Neon H279 ki/ki cells (left) and upon EMT induction by TGFβ treatment for 0-3 (middle) and 6-10 days. Bright-field and green channels are shown as overlay (upper) and green fluorescence channel alone (lower panels) imaged every 2 hours (Incucyte S3), showing high levels of Neon fluorescence that are not changing during TGFβ treatment (see also Fig. 5A,C). Scale (time-stamp box), 100 µm.

Video S9. Composite video from individual time-lapse recordings of live cell imaging of untreated epithelial KPC;Zeb1-Neon G623 ki/ki cells (left) and upon EMT induction by TGFβ treatment for 0-4 (middle) and 6-10 days. Bright-field and green channels are shown as overlay

(upper) and green fluorescence channel alone (lower panels) imaged every 2 hours (Incucyte S3), showing moderate levels of Neon fluorescence that are increasing during 0-4 d TGFβ treatment (see also Fig. 5A,B). Scale (time-stamp box), 100 μm.

Video S10. Composite video from individual time-lapse recordings of live cell imaging of untreated epithelial KPC;Zeb1-Neon G975 ki/+ cells (left) and upon EMT induction by TGFβ treatment for 0-4 (middle) and 6-10 days. Bright-field and green channels are shown as overlay (upper) and green fluorescence channel alone (lower panels) imaged every 2 hours (Incucyte S3), showing low levels of Neon fluorescence that are increasing during 0-4 d TGFβ treatment (see also Fig. 5A). Scale (time-stamp box), 100 μm.
