# Supplementary figures and images for: A ZEB1-Neon knock-in uncovers traceable dynamics of epithelial-mesenchymal transition in tumors in vivo
Source: BMC Biol. 2026 May 12;24:116. doi: 10.1186/s12915-026-02629-0 (PMC13170155; doi:10.1186/s12915-026-02629-0)

Fig. 1B

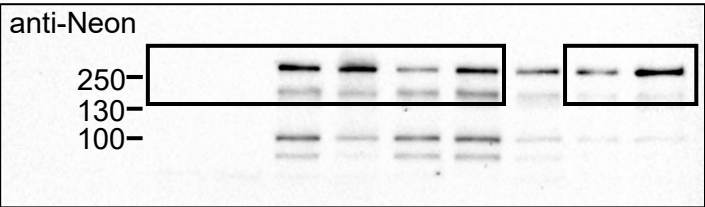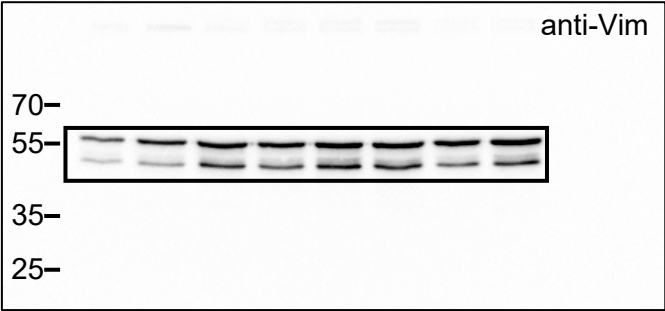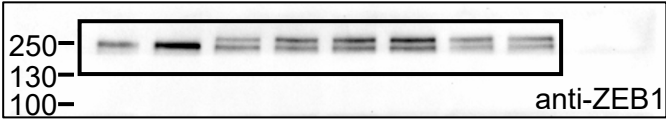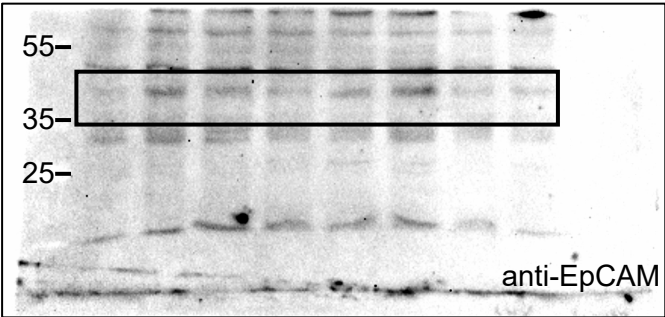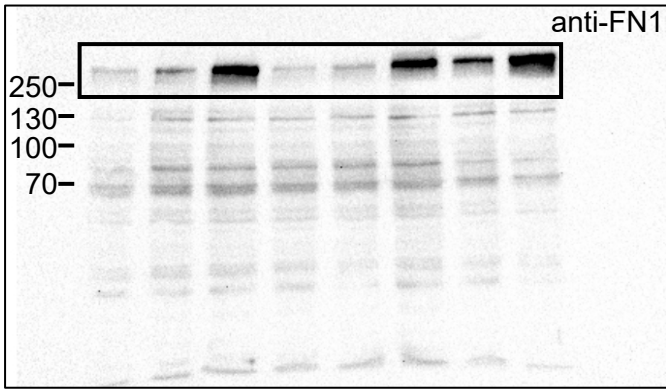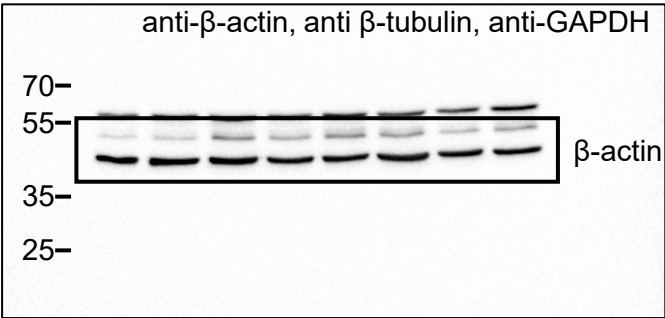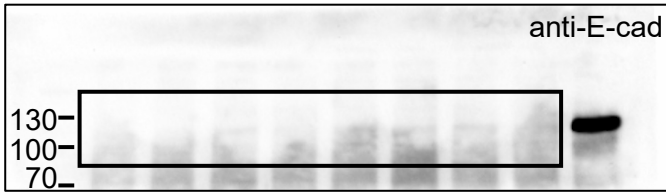

Fig. 1F

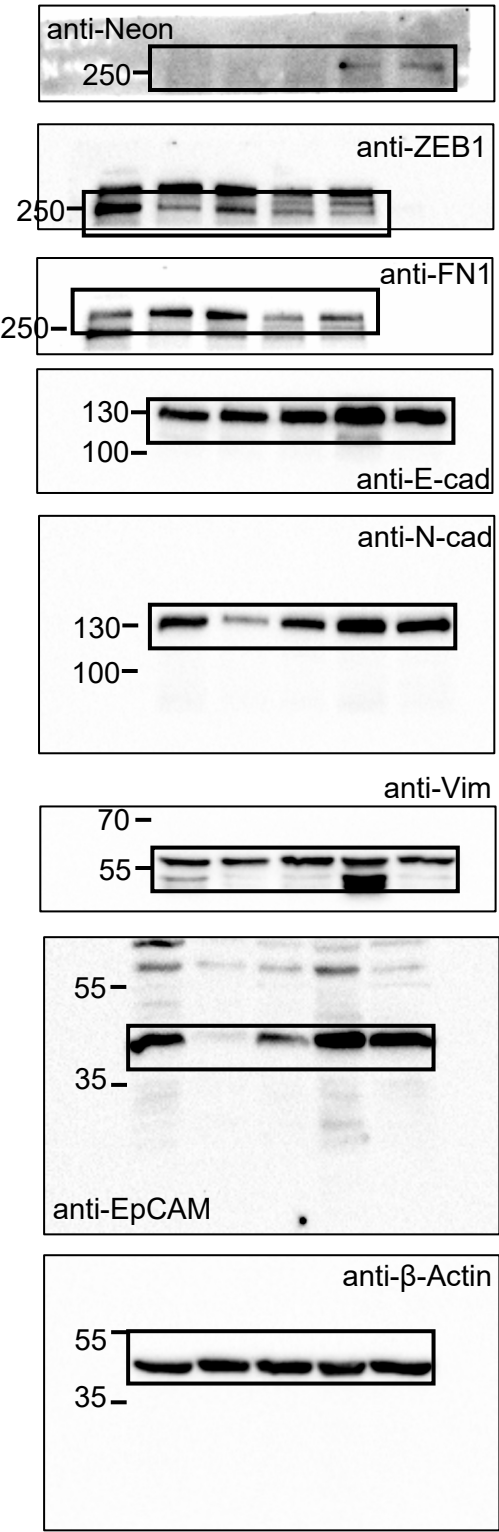

Fig. 1G

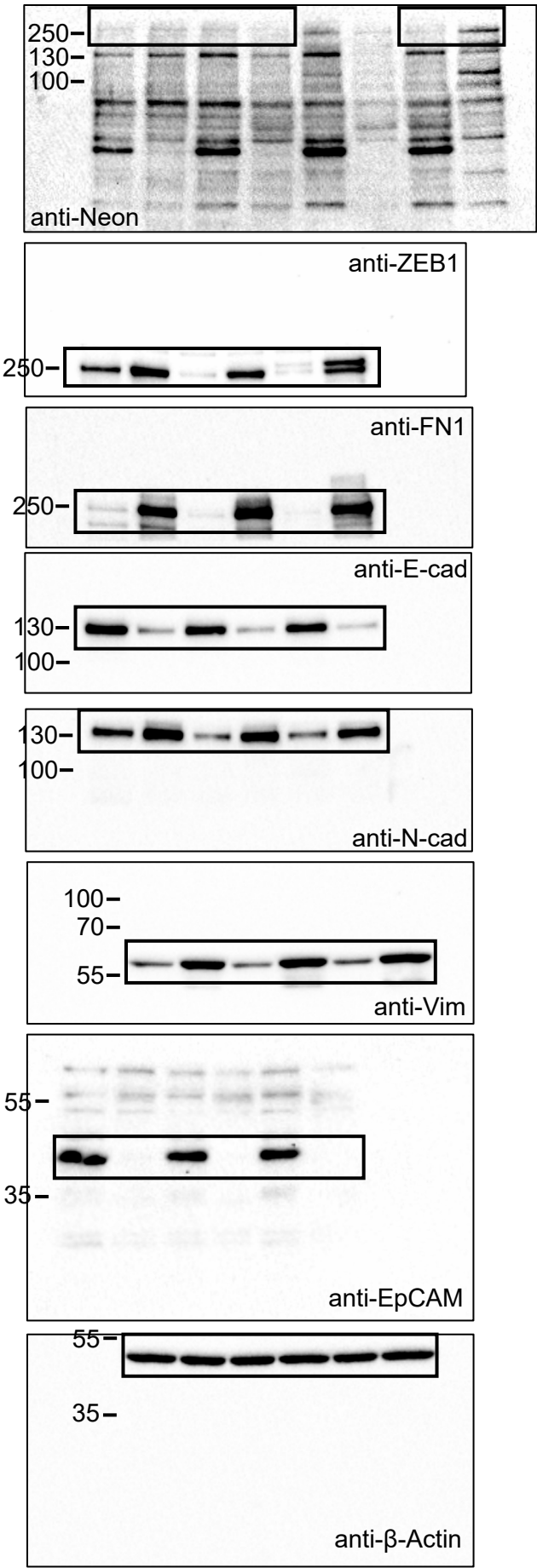

Fig. 2B

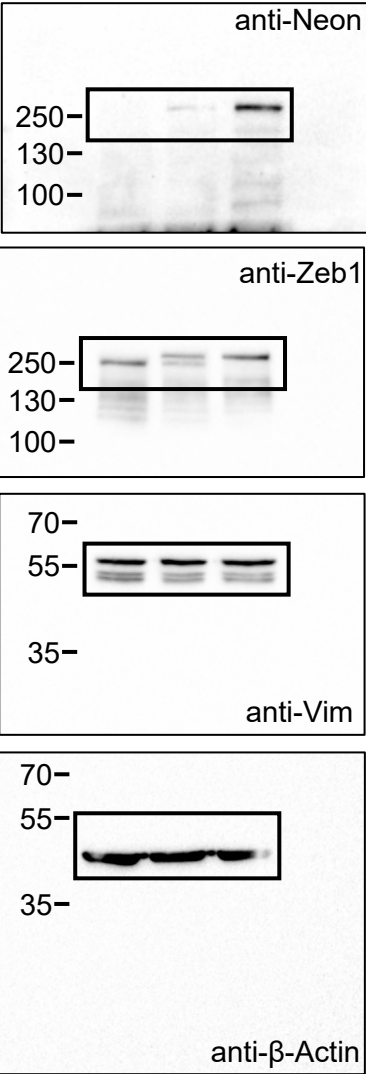

Fig. 4A

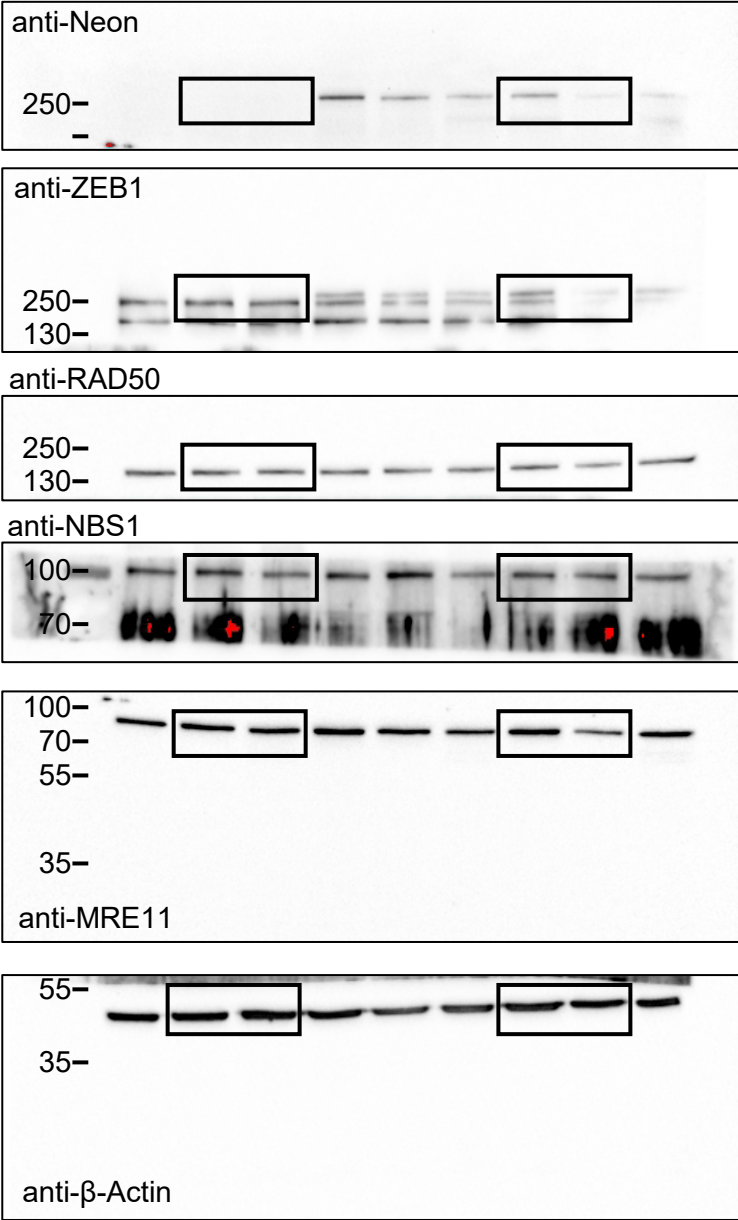

Fig. 4B

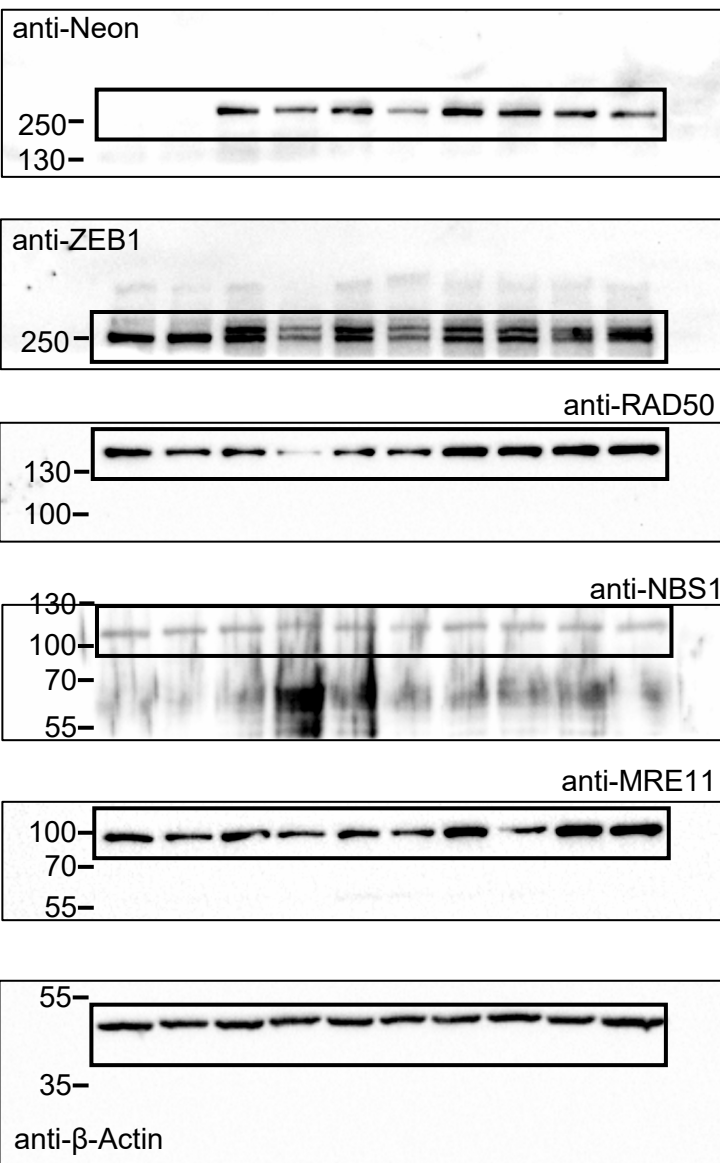

Fig. 4C

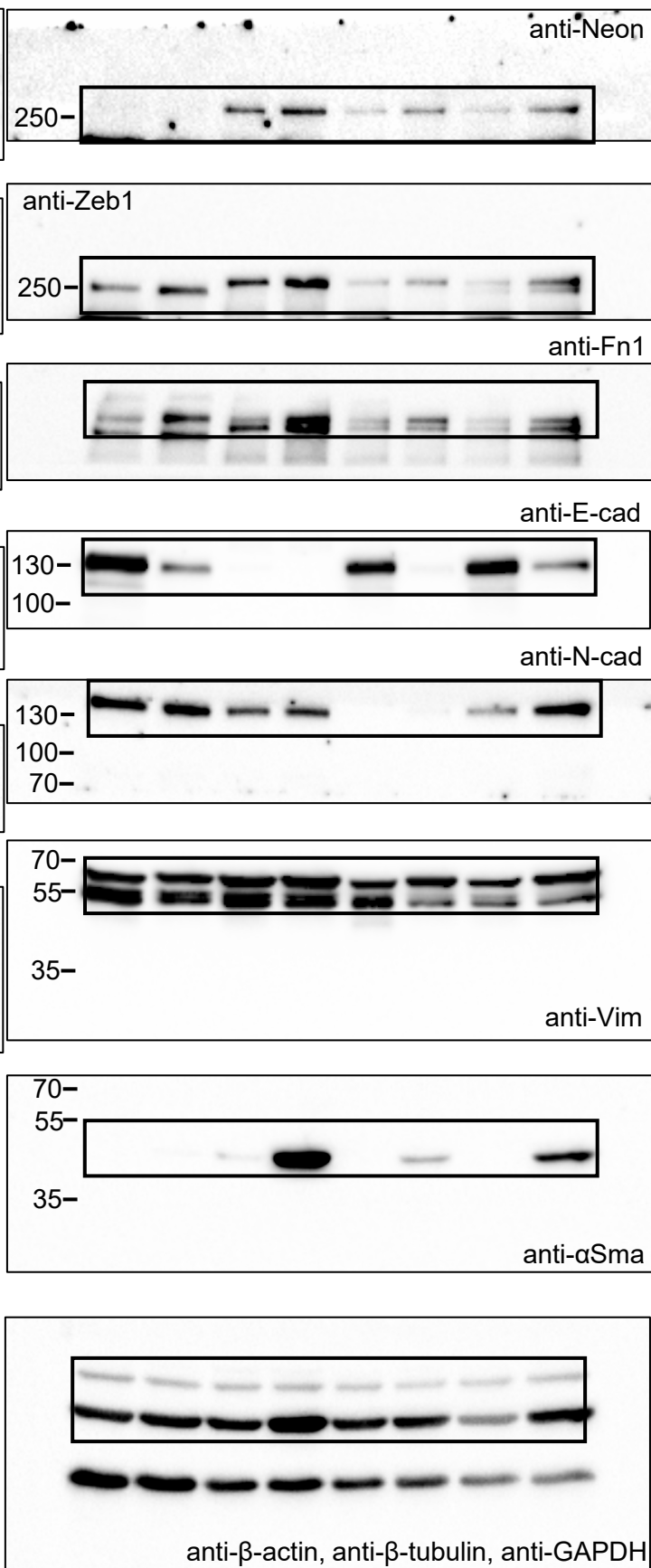

Suppl. Fig. S4B

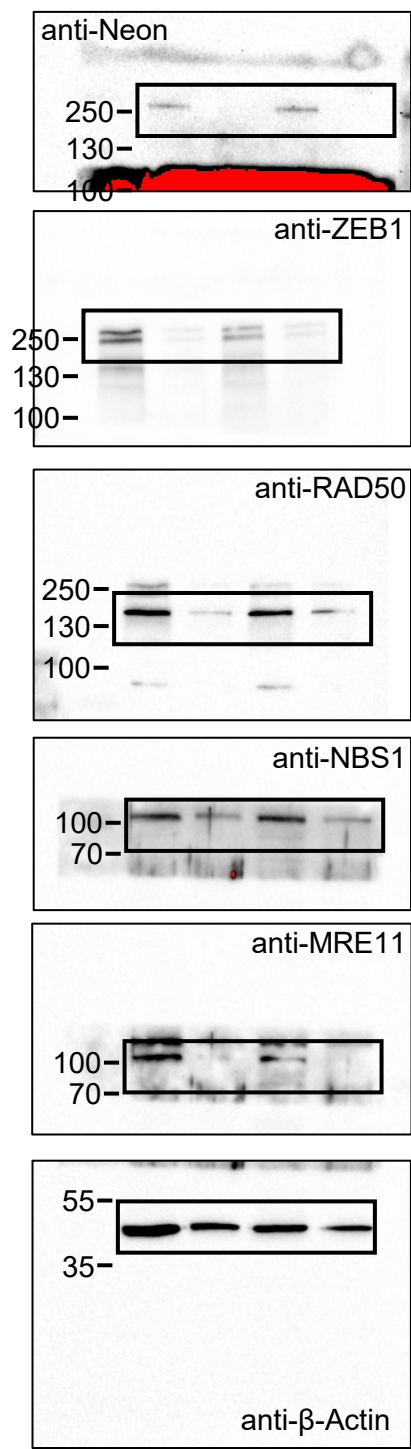

Suppl. Fig. S4D

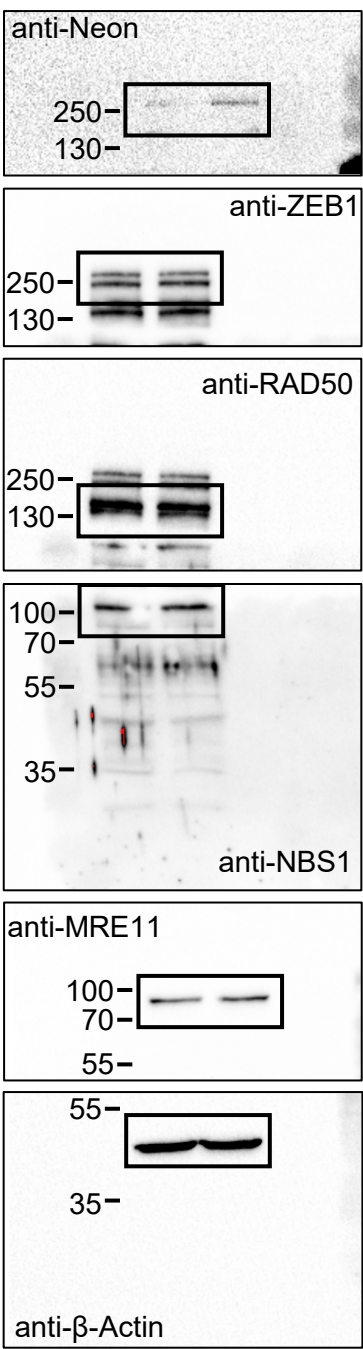

Suppl. Fig. S5B

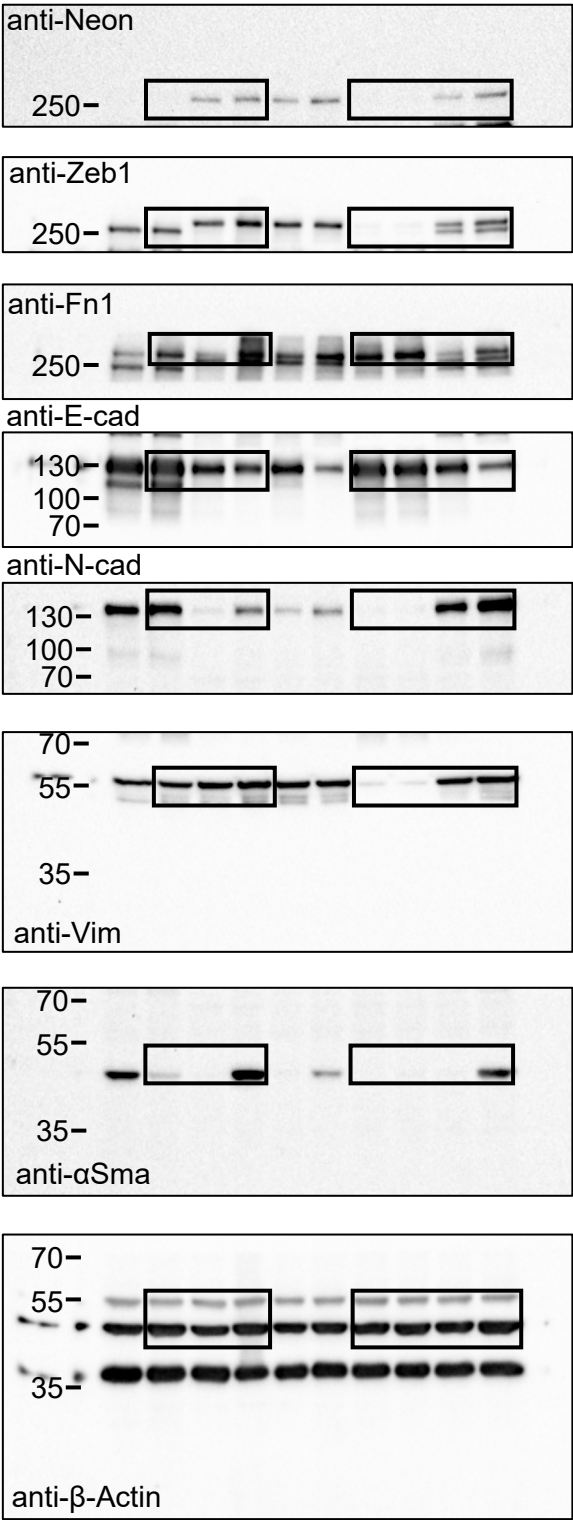

Supplement: Supplementary file 4 — Additional file 4. [file 12915_2026_2629_MOESM4_ESM.pdf]
